# Supplementary material for: Standard multiscale entropy reflects neural dynamics at mismatched temporal scales: What’s signal irregularity got to do with it?
Source: PLoS Comput Biol. 2020 May 11;16(5):e1007885. doi: 10.1371/journal.pcbi.1007885 (PMC7241858; doi:10.1371/journal.pcbi.1007885)
Supplement: S1 Text — (PDF) [file pcbi.1007885.s001.pdf]

Supplementary Information for

Standard multiscale entropy reflects neural dynamics at mismatched temporal scales

Julian Q. Kosciessa, Niels A. Kloosterman, and Douglas D. Garrett

Email: kosciessa@mpib-berlin.mpg.de; garrett@mpib-berlin.mpg.de

S1 Text. Systematic literature search assessing the prevalence of global similarity bounds.

We performed a systematic literature search to assess the prevalence of global similarity bounds in current neuroscientific applications (heart rate variability applications are specifically marked). We searched Pubmed (https://www.ncbi.nlm.nih.gov/pubmed) with the following terms: *(MSE AND sample entropy AND EEG) OR (MSE AND brain AND variability) OR (MSE AND EEG AND variability) OR (multiscale entropy AND EEG AND variability).* We excluded any studies that did not assess multiscale entropy, including studies that were restricted to sample entropy at scale 1. In addition, we added references from the main text that were not captured by the systematic search (highlighted in grey). For MSE applications, we checked the text for a notion of how similarity bounds were computed, i.e., whether it was calculated as r*SD of the original time series or the coarse-grained time series. The following sections list the results of this qualitative review and is purely intended to characterize the prevalence of global similarity bounds, not as a qualitative judgement on the claims made in any particular paper. Our literature search revealed the following papers. The relative amount of studies with presumably global similarity bounds was as follows (39+13)/(39+13+4) = 0,928; i.e., > 90%.

Scale-invariant similarity bounds (r x global SD)
We chose this category, when the article contained the specific information that r was calculated from the original signal (i.e., scale-invariant).

Azami, Fernandez, and Escudero (2017)
Azami, Rostaghi, Abasolo, and Escudero (2017)
Carpentier et al. (2019)
Escudero, Abasolo, Hornero, Espino, and Lopez (2006) [but they note the issue]
Grandy, Garrett, Schmiedek, and Werkle-Bergner (2016)
Hadoush, Alafeef, and Abdulhay (2019)
Kaur et al. (2019)
M. Liu, Song, Liang, Knopfel, and Zhou (2019)
H. Liu et al. (2017) [HRV]
Lu et al. (2015)
McIntosh, Kovacevic, and Itier (2008)
Mizuno et al. (2010)
Weng et al. (2015)

#: 13

Unclear, assumed scale-invariant similarity bounds (r x global SD)
We chose this category, when the article did not contain any information about how r was calculated, or no reference was made to scale-specific adaptations. For many papers, Costa, Goldberger, and Peng (2002, 2005) or Richman and Moorman (2000) were cited, which use scale-invariant implementations.

Raja Beharelle, Kovacevic, McIntosh, and Levine (2012)
Bertrand et al. (2016)
Catarino, Churches, Baron-Cohen, Andrade, and Ring (2011)
Chen et al. (2015)(HRV)

Chen et al. (2018) (HRV)
Li, Chen, Li, Wang, and Liu (2016)
Chiu et al. (2015) (HRV)
Courtiol et al. (2016)
Gao, Hu, Liu, and Cao (2015)
Harati, Crowell, Huang, Mayberg, and Nemati (2019)
Harati, Crowell, Mayberg, Jun, and Nemati (2016)
Hasegawa et al. (2018)
Heisz and McIntosh (2013)
Heisz, Shedden, and McIntosh (2012)
Hu and Liang (2012) [RM]
Hussain, Saeed, Awan, and Idris (2018)
Hussain, Aziz, et al. (2018)
Jaworska et al. (2018)
Kuntzelman, Jack Rhodes, Harrington, and Miskovic (2018)
Lin et al. (2019) [BOLD]
H. Liu et al. (2018)
H. Y. Liu et al. (2018)
Q. Liu, Chen, Fan, Abbod, and Shieh (2015)
Q. Liu, Chen, Fan, Abbod, and Shieh (2017)
McIntosh et al. (2014)
Misic et al. (2015)
Misic, Vakorin, Paus, and McIntosh (2011)
Miskovic, Owens, Kuntzelman, and Gibb (2016)
Park, Kim, Kim, Cichocki, and Kim (2007)
Roldan, Molina-Pico, Cuesta-Frau, Martinez, and Crespo (2011)
Szostakiwskyj, Willatt, Cortese, and Protzner (2017)
Takahashi et al. (2009)
Takahashi et al. (2010)
Takahashi et al. (2016)
Ueno et al. (2015)
Yang et al. (2013)
H. Y. Wang, McIntosh, Kovacevic, Karachalios, and Protzner (2016)
H. Wang, Pexman, Turner, Cortese, and Protzner (2018)
Wei et al. (2014)

#: 39

Scale-wise similarity bounds (r x scale-wise SD)

We chose this category, when the article either specified that scale-wise recalculation of r parameters was performed, or when the description could allow that inference.

Fabris et al. (2014) [but with unclear variations in r]
Sleimen-Malkoun et al. (2015)
Valencia et al. (2009) [HRV]
Zavala-Yoe, Ramirez-Mendoza, and Cordero (2015)

#: 4

Not applicable

We chose this category, when multi-scale entropy was not used in the study (i.e., erroneous listing of paper).

El-Gohary, McNames, and Elsas (2008)
Erdogan, Yucel, and Akin (2014)
Fernandez, Gomez, Hornero, and Lopez-Ibor (2013)
Heunis, Aldrich, and de Vries (2016)
Hier, Jao, and Brint (1994)
Kielar et al. (2016) [BOLD MSE, single scale]
Nazari et al. (2019)
Puce, Berkovic, Cadusch, and Bladin (1994)
Sinai, Phillips, Chertkow, and Kabani (2010)
Verhaeghe, Gravel, and Reader (2010)
Xu, Cui, Hong, and Liang (2015)

Supplementary References

Azami, H., Fernandez, A., & Escudero, J. (2017). Refined multiscale fuzzy entropy based on standard deviation for biomedical signal analysis. *Med Biol Eng Comput, 55*(11), 2037-2052. doi:10.1007/s11517-017-1647-5

Azami, H., Rostaghi, M., Abasolo, D., & Escudero, J. (2017). Refined Composite Multiscale Dispersion Entropy and its Application to Biomedical Signals. *Ieee Transactions on Biomedical Engineering, 64*(12), 2872-2879. doi:10.1109/tbme.2017.2679136

Bandt, C., & Pompe, B. (2002). Permutation entropy: A natural complexity measure for time series. *Physical Review Letters, 88*(17). doi:10.1103/PhysRevLett.88.174102

Bertrand, J. A., McIntosh, A. R., Postuma, R. B., Kovacevic, N., Latreille, V., Panisset, M., . . . Gagnon, J. F. (2016). Brain Connectivity Alterations Are Associated with the Development of Dementia in Parkinson's Disease. *Brain Connectivity, 6*(3), 216-224. doi:10.1089/brain.2015.0390

Carpentier, S. M., McCulloch, A. R., Brown, T. M., Ritter, P., Wang, Z., Salimpoor, V., . . . McIntosh, A. R. (2019). Complexity matching: brain signals mirror environment information patterns during music listening and reward. *bioRxiv*.

Catarino, A., Churches, O., Baron-Cohen, S., Andrade, A., & Ring, H. (2011). Atypical EEG complexity in autism spectrum conditions: A multiscale entropy analysis. *Clinical Neurophysiology, 122*(12), 2375-2383. doi:10.1016/j.clinph.2011.05.004

Chen, C. H., Huang, P. W., Tang, S. C., Shieh, J. S., Lai, D. M., Wu, A. Y., & Jeng, J. S. (2015). Complexity of Heart Rate Variability Can Predict Stroke-In-Evolution in Acute Ischemic Stroke Patients. *Sci Rep, 5*, 17552. doi:10.1038/srep17552

Chen, C. H., Tang, S. C., Lee, D. Y., Shieh, J. S., Lai, D. M., Wu, A. Y., & Jeng, J. S. (2018). Impact of Supratentorial Cerebral Hemorrhage on the Complexity of Heart Rate Variability in Acute Stroke. *Sci Rep, 8*(1), 11473. doi:10.1038/s41598-018-29961-y

Chiu, H. C., Lin, Y. H., Lo, M. T., Tang, S. C., Wang, T. D., Lu, H. C., . . . Peng, C. K. (2015). Complexity of cardiac signals for predicting changes in alpha-waves after stress in patients undergoing cardiac catheterization. *Scientific Reports, 5*. doi:10.1038/srep13315

Costa, M., Goldberger, A. L., & Peng, C. K. (2002). Multiscale entropy analysis of complex physiologic time series. *Physical Review Letters, 89*(6). doi:10.1103/PhysRevLett.89.068102

Costa, M., Goldberger, A. L., & Peng, C. K. (2005). Multiscale entropy analysis of biological signals. *Physical Review E, 71*(2). doi:10.1103/PhysRevE.71.021906

Courtiol, J., Perdikis, D., Petkoski, S., Muller, V., Huys, R., Sleimen-Malkoun, R., & Jirsa, V. K. (2016). The multiscale entropy: Guidelines for use and interpretation in brain signal analysis. *Journal of Neuroscience Methods, 273*, 175-190. doi:10.1016/j.jneumeth.2016.09.004

El-Gohary, M., McNames, J., & Elsas, S. (2008). User-guided interictal spike detection. *Conf Proc IEEE Eng Med Biol Soc, 2008*, 821-824. doi:10.1109/iembs.2008.4649280

Erdogan, S. B., Yucel, M. A., & Akin, A. (2014). Analysis of task-evoked systemic interference in fNIRS measurements: insights from fMRI. *Neuroimage, 87*, 490-504. doi:10.1016/j.neuroimage.2013.10.024

Escudero, J., Abasolo, D., Hornero, R., Espino, P., & Lopez, M. (2006). Analysis of electroencephalograms in Alzheimer's disease patients with multiscale entropy. *Physiological Measurement, 27*(11), 1091-1106. doi:10.1088/0967-3334/27/11/004

Fabris, C., Sparacino, G., Sejling, A. S., Goljahani, A., Duun-Henriksen, J., Remvig, L. S., . . . Cobelli, C. (2014). Hypoglycemia-Related Electroencephalogram Changes Assessed by Multiscale Entropy. *Diabetes Technology & Therapeutics, 16*(10), 688-694. doi:10.1089/dia.2013.0331

Fernandez, A., Gomez, C., Hornero, R., & Lopez-Ibor, J. J. (2013). Complexity and schizophrenia. *Prog Neuropsychopharmacol Biol Psychiatry, 45*, 267-276. doi:10.1016/j.pnpbp.2012.03.015

Gao, J., Hu, J., Liu, F., & Cao, Y. (2015). Multiscale entropy analysis of biological signals: a fundamental bi-scaling law. *Front Comput Neurosci, 9*, 64. doi:10.3389/fncom.2015.00064

Grandy, T. H., Garrett, D. D., Schmiedek, F., & Werkle-Bergner, M. (2016). On the estimation of brain signal entropy from sparse neuroimaging data. *Scientific Reports, 6*. doi:10.1038/srep23073

Hadoush, H., Alafeef, M., & Abdulhay, E. (2019). Brain Complexity in Children with Mild and Severe Autism Spectrum Disorders: Analysis of Multiscale Entropy in EEG. *Brain Topogr*. doi:10.1007/s10548-019-00711-1

Harati, S., Crowell, A., Huang, Y., Mayberg, H., & Nemati, S. (2019). Classifying Depression Severity in Recovery from Major Depressive Disorder via Dynamic Facial Features. *IEEE J Biomed Health Inform*. doi:10.1109/jbhi.2019.2930604

Harati, S., Crowell, A., Mayberg, H., Jun, K., & Nemati, S. (2016). Discriminating clinical phases of recovery from major depressive disorder using the dynamics of facial expression. *Conf Proc IEEE Eng Med Biol Soc, 2016*, 2254-2257. doi:10.1109/embc.2016.7591178

Hasegawa, C., Takahashi, T., Yoshimura, Y., Nobukawa, S., Ikeda, T., Saito, D. N., . . . Kikuchi, M. (2018). Developmental Trajectory of Infant Brain Signal Variability: A Longitudinal Pilot Study. *Front Neurosci, 12*, 566. doi:10.3389/fnins.2018.00566
Heisz, J. J., & McIntosh, A. R. (2013). Applications of EEG Neuroimaging Data: Event-related Potentials, Spectral Power, and Multiscale Entropy. *Jove-Journal of Visualized Experiments*(76). doi:10.3791/50131
Heisz, J. J., Shedden, J. M., & McIntosh, A. R. (2012). Relating brain signal variability to knowledge representation. *Neuroimage, 63*(3), 1384-1392. doi:10.1016/j.neuroimage.2012.08.018
Heunis, T. M., Aldrich, C., & de Vries, P. J. (2016). Recent Advances in Resting-State Electroencephalography Biomarkers for Autism Spectrum Disorder-A Review of Methodological and Clinical Challenges. *Pediatr Neurol, 61*, 28-37. doi:10.1016/j.pediatrneurol.2016.03.010
Hier, D. B., Jao, C. S., & Brint, S. U. (1994). The Mental Status Expert (MSE): an expert system for scoring and interpreting the mental status examination. *Proc Annu Symp Comput Appl Med Care*, 1053.
Hu, M., & Liang, H. (2012). Adaptive multiscale entropy analysis of multivariate neural data. *IEEE Trans Biomed Eng, 59*(1), 12-15. doi:10.1109/tbme.2011.2162511
Hussain, L., Aziz, W., Saeed, S., Shah, S. A., Nadeem, M. S. A., Awan, I. A., . . . Kazmi, S. Z. H. (2018). Quantifying the dynamics of electroencephalographic (EEG) signals to distinguish alcoholic and non-alcoholic subjects using an MSE based K-d tree algorithm. *Biomed Tech (Berl), 63*(4), 481-490. doi:10.1515/bmt-2017-0041
Hussain, L., Saeed, S., Awan, I. A., & Idris, A. (2018). Multiscaled Complexity Analysis of EEG Epileptic Seizure Using Entropy-Based Techniques. *Archives of Neuroscience, 5*(1). doi:10.5812/archneurosci.61161
Jaworska, N., Wang, H., Smith, D. M., Blier, P., Knott, V., & Protzner, A. B. (2018). Pre-treatment EEG signal variability is associated with treatment success in depression. *Neuroimage Clin, 17*, 368-377. doi:10.1016/j.nicl.2017.10.035
Kaur, Y., Ouyang, G., Junge, M., Sommer, W., Liu, M., Zhou, C., & Hildebrandt, A. (2019). The reliability and psychometric structure of Multi-Scale Entropy measured from EEG signals at rest and during face and object recognition tasks. *J Neurosci Methods, 326*, 108343. doi:10.1016/j.jneumeth.2019.108343
Kielar, A., Deschamps, T., Chu, R. K., Jokel, R., Khatamian, Y. B., Chen, J. J., & Meltzer, J. A. (2016). Identifying Dysfunctional Cortex: Dissociable Effects of Stroke and Aging on Resting State Dynamics in MEG and fMRI. *Front Aging Neurosci, 8*, 40. doi:10.3389/fnagi.2016.00040
Kuntzelman, K., Jack Rhodes, L., Harrington, L. N., & Miskovic, V. (2018). A practical comparison of algorithms for the measurement of multiscale entropy in neural time series data. *Brain Cogn, 123*, 126-135. doi:10.1016/j.bandc.2018.03.010
Li, C. X., Chen, Y. N., Li, Y. J., Wang, J., & Liu, T. (2016). Complexity analysis of brain activity in attention-deficit/hyperactivity disorder: A multiscale entropy analysis. *Brain Research Bulletin, 124*, 12-20. doi:10.1016/j.brainresbull.2016.03.007
Lin, C., Lee, S. H., Huang, C. M., Chen, G. Y., Ho, P. S., Liu, H. L., . . . Wu, S. C. (2019). Increased brain entropy of resting-state fMRI mediates the relationship between depression severity and mental health-related quality of life in late-life depressed elderly. *J Affect Disord, 250*, 270-277. doi:10.1016/j.jad.2019.03.012
Liu, H., Yang, Z., Meng, F., Guan, Y., Ma, Y., Liang, S., . . . Li, L. (2017). Impairment of heart rhythm complexity in patients with drug-resistant epilepsy: An assessment with multiscale entropy analysis. *Epilepsy Research, 138*, 11-17. doi:10.1016/j.eplepsyres.2017.10.002
Liu, H., Yang, Z., Meng, F., Huang, L., Qu, W., Hao, H., . . . Li, L. (2018). Chronic vagus nerve stimulation reverses heart rhythm complexity in patients with drug-resistant epilepsy: An assessment with multiscale entropy analysis. *Epilepsy Behav, 83*, 168-174. doi:10.1016/j.yebeh.2018.03.035
Liu, H. Y., Yang, Z., Meng, F. G., Guan, Y. G., Ma, Y. S., Liang, S. L., . . . Li, L. M. (2018). Preoperative Heart Rate Variability as Predictors of Vagus Nerve Stimulation Outcome in Patients with Drug-resistant Epilepsy. *Sci Rep, 8*(1), 3856. doi:10.1038/s41598-018-21669-3
Liu, M., Song, C., Liang, Y., Knopfel, T., & Zhou, C. (2019). Assessing spatiotemporal variability of brain spontaneous activity by multiscale entropy and functional connectivity. *Neuroimage, 198*, 198-220. doi:10.1016/j.neuroimage.2019.05.022
Liu, Q., Chen, Y. F., Fan, S. Z., Abbod, M. F., & Shieh, J. S. (2015). EEG Signals Analysis Using Multiscale Entropy for Depth of Anesthesia Monitoring during Surgery through Artificial Neural Networks. *Computational and Mathematical Methods in Medicine*. doi:10.1155/2015/232381
Liu, Q., Chen, Y. F., Fan, S. Z., Abbod, M. F., & Shieh, J. S. (2017). EEG artifacts reduction by multivariate empirical mode decomposition and multiscale entropy for monitoring depth of

anaesthesia during surgery. *Medical & Biological Engineering & Computing, 55*(8), 1435-1450. doi:10.1007/s11517-016-1598-2
Lu, W. Y., Chen, J. Y., Chang, C. F., Weng, W. C., Lee, W. T., & Shieh, J. S. (2015). Multiscale Entropy of Electroencephalogram as a Potential Predictor for the Prognosis of Neonatal Seizures. *Plos One, 10*(12). doi:10.1371/journal.pone.0144732
McIntosh, A. R., Kovacevic, N., & Itier, R. J. (2008). Increased Brain Signal Variability Accompanies Lower Behavioral Variability in Development. *Plos Computational Biology, 4*(7). doi:10.1371/journal.pcbi.1000106
McIntosh, A. R., Vakorin, V., Kovacevic, N., Wang, H., Diaconescu, A., & Protzner, A. B. (2014). Spatiotemporal Dependency of Age-Related Changes in Brain Signal Variability. *Cerebral Cortex, 24*(7), 1806-1817. doi:10.1093/cercor/bht030
Misic, B., Doesburg, S. M., Fatima, Z., Vidal, J., Vakorin, V. A., Taylor, M. J., & McIntosh, A. R. (2015). Coordinated Information Generation and Mental Flexibility: Large-Scale Network Disruption in Children with Autism. *Cerebral Cortex, 25*(9), 2815-2827. doi:10.1093/cercor/bhu082
Misic, B., Vakorin, V. A., Paus, T., & McIntosh, A. R. (2011). Functional embedding predicts the variability of neural activity. *Frontiers in Systems Neuroscience, 5*, 90. doi:10.3389/fnsys.2011.00090
Miskovic, V., Owens, M., Kuntzelman, K., & Gibb, B. E. (2016). Charting moment-to-moment brain signal variability from early to late childhood. *Cortex, 83*, 51-61. doi:10.1016/j.cortex.2016.07.006
Mizuno, T., Takahashi, T., Cho, R. Y., Kikuchi, M., Murata, T., Takahashi, K., & Wada, Y. (2010). Assessment of EEG dynamical complexity in Alzheimer's disease using multiscale entropy. *Clinical Neurophysiology, 121*(9), 1438-1446. doi:10.1016/j.clinph.2010.03.025
Nazari, A., Alavimajd, H., Shakeri, N., Bakhshandeh, M., Faghihzadeh, E., & Marzbani, H. (2019). Prediction of Brain Connectivity Map in Resting-State fMRI Data Using Shrinkage Estimator. *Basic Clin Neurosci, 10*(2), 147-156. doi:10.32598/bcn.9.10.140
Ouyang, G. X., Li, J., Liu, X. Z., & Li, X. L. (2013). Dynamic characteristics of absence EEG recordings with multiscale permutation entropy analysis. *Epilepsy Research, 104*(3), 246-252. doi:10.1016/j.eplepsyres.2012.11.003
Park, J. H., Kim, S., Kim, C. H., Cichocki, A., & Kim, K. (2007). Multiscale entropy analysis of EEG from patients under different pathological conditions. *Fractals-Complex Geometry Patterns and Scaling in Nature and Society, 15*(4), 399-404. doi:10.1142/S0218348x07003691
Puce, A., Berkovic, S. F., Cadusch, P. J., & Bladin, P. F. (1994). P3 latency jitter assessed using 2 techniques. I. Simulated data and surface recordings in normal subjects. *Electroencephalogr Clin Neurophysiol, 92*(4), 352-364. doi:10.1016/0168-5597(94)90103-1
Raja Beharelle, A., Kovacevic, N., McIntosh, A. R., & Levine, B. (2012). Brain signal variability relates to stability of behavior after recovery from diffuse brain injury. *Neuroimage, 60*(2), 1528-1537. doi:10.1016/j.neuroimage.2012.01.037
Richman, J. S., & Moorman, J. R. (2000). Physiological time-series analysis using approximate entropy and sample entropy. *American Journal of Physiology-Heart and Circulatory Physiology, 278*(6), H2039-H2049.
Riedl, M., Muller, A., & Wessel, N. (2013). Practical considerations of permutation entropy. *European Physical Journal-Special Topics, 222*(2), 249-262. doi:10.1140/epjst/e2013-01862-7
Roldan, E. M., Molina-Pico, A., Cuesta-Frau, D., Martinez, P. M., & Crespo, S. O. (2011). Characterization of entropy measures against data loss: application to EEG records. *Conf Proc IEEE Eng Med Biol Soc, 2011*, 6110-6113. doi:10.1109/iembs.2011.6091509
Sinai, M., Phillips, N. A., Chertkow, H., & Kabani, N. J. (2010). Task switching performance reveals heterogeneity amongst patients with mild cognitive impairment. *Neuropsychology, 24*(6), 757-774. doi:10.1037/a0020314
Sleimen-Malkoun, R., Perdikis, D., Muller, V., Blanc, J. L., Huys, R., Temprado, J. J., & Jirsa, V. K. (2015). Brain Dynamics of Aging: Multiscale Variability of EEG Signals at Rest and during an Auditory Oddball Task(1,2,3). *Eneuro, 2*(3). doi:10.1523/ENEURO.0067-14.2015
Szostakiwskyj, J. M. H., Willatt, S. E., Cortese, F., & Protzner, A. B. (2017). The modulation of EEG variability between internally-and externally-driven cognitive states varies with maturation and task performance. *Plos One, 12*(7). doi:10.1371/journal.pone.0181894
Takahashi, T., Cho, R., Mizuno, T., Kikuchi, M., Murata, T., Takahashi, K., & Wada, Y. (2010). Antipsychotics reverse abnormal EEG complexity in drug-naive schizophrenia: A multiscale entropy analysis. *International Journal of Neuropsychopharmacology, 13*, 242-243.
Takahashi, T., Cho, R. Y., Murata, T., Mizuno, T., Kikuchi, M., Mizukami, K., . . . Wada, Y. (2009). Age-related variation in EEG complexity to photic stimulation: A multiscale entropy analysis. *Clinical Neurophysiology, 120*(3), 476-483. doi:10.1016/j.clinph.2008.12.043

Takahashi, T., Yoshimura, Y., Hiraishi, H., Hasegawa, C., Munesue, T., Higashida, H., . . . Kikuchi, M. (2016). Enhanced brain signal variability in children with autism spectrum disorder during early childhood. *Human Brain Mapping, 37*(3), 1038-1050. doi:10.1002/hbm.23089

Ueno, K., Takahashi, T., Takahashi, K., Mizukami, K., Tanaka, Y., & Wada, Y. (2015). Neurophysiological basis of creativity in healthy elderly people: A multiscale entropy approach. *Clinical Neurophysiology, 126*(3), 524-531. doi:10.1016/j.clinph.2014.06.032

Valencia, J. F., Porta, A., Vallverdu, M., Claria, F., Baranowski, R., Orlowska-Baranowska, E., & Caminal, P. (2009). Refined Multiscale Entropy: Application to 24-h Holter Recordings of Heart Period Variability in Healthy and Aortic Stenosis Subjects. *Ieee Transactions on Biomedical Engineering, 56*(9), 2202-2213. doi:10.1109/Tbme.2009.2021986

Verhaeghe, J., Gravel, P., & Reader, A. J. (2010). Task-oriented quantitative image reconstruction in emission tomography for single- and multi-subject studies. *Phys Med Biol, 55*(23), 7263-7285. doi:10.1088/0031-9155/55/23/006

Wang, H., Pexman, P. M., Turner, G., Cortese, F., & Protzner, A. B. (2018). The relation between Scrabble expertise and brain aging as measured with EEG brain signal variability. *Neurobiology of Aging, 69*, 249-260. doi:10.1016/j.neurobiolaging.2018.05.015

Wang, H. Y., McIntosh, A. R., Kovacevic, N., Karachalios, M., & Protzner, A. B. (2016). Age-related Multiscale Changes in Brain Signal Variability in Pre-task versus Post-task Resting-state EEG. *Journal of Cognitive Neuroscience, 28*(7), 971-984. doi:10.1162/jocn_a_00947

Wei, Q., Li, Y., Fan, S. Z., Liu, Q., Abbod, M. F., Lu, C. W., . . . Shieh, J. S. (2014). A critical care monitoring system for depth of anaesthesia analysis based on entropy analysis and physiological information database. *Australasian Physical & Engineering Sciences in Medicine, 37*(3), 591-605. doi:10.1007/s13246-014-0285-6

Weng, W. C., Jiang, G. J., Chang, C. F., Lu, W. Y., Lin, C. Y., Lee, W. T., & Shieh, J. S. (2015). Complexity of Multi-Channel Electroencephalogram Signal Analysis in Childhood Absence Epilepsy. *Plos One, 10*(8), e0134083. doi:10.1371/journal.pone.0134083

Xu, Y., Cui, J., Hong, W., & Liang, H. (2015). [Automatic Classification of Epileptic Electroencephalogram Signal Based on Improved Multivariate Multiscale Entropy]. *Sheng Wu Yi Xue Gong Cheng Xue Za Zhi, 32*(2), 256-262.

Yang, A. C., Wang, S. J., Lai, K. L., Tsai, C. F., Yang, C. H., Hwang, J. P., . . . Fuh, J. L. (2013). Cognitive and neuropsychiatric correlates of EEG dynamic complexity in patients with Alzheimer's disease. *Progress in Neuro-Psychopharmacology & Biological Psychiatry, 47*, 52-61. doi:10.1016/j.pnpbp.2013.07.022

Zanin, M., Zunino, L., Rosso, O. A., & Papo, D. (2012). Permutation Entropy and Its Main Biomedical and Econophysics Applications: A Review. *Entropy, 14*(8), 1553-1577. doi:10.3390/e14081553

Zavala-Yoe, R., Ramirez-Mendoza, R., & Cordero, L. M. (2015). Novel way to investigate evolution of children refractory epilepsy by complexity metrics in massive information. *Springerplus, 4*. doi:10.1186/s40064-015-1173-6
