# Supplementary material for: Standard multiscale entropy reflects neural dynamics at mismatched temporal scales: What’s signal irregularity got to do with it?
Source: PLoS Comput Biol. 2020 May 11;16(5):e1007885. doi: 10.1371/journal.pcbi.1007885 (PMC7241858; doi:10.1371/journal.pcbi.1007885)
Supplement: S2 Text — (PDF) [file pcbi.1007885.s002.pdf]

Supplementary Information for

Standard multiscale entropy reflects neural dynamics at mismatched temporal scales

Julian Q. Kosciessa, Niels A. Kloosterman, and Douglas D. Garrett

Email: kosciessa@mpib-berlin.mpg.de; garrett@mpib-berlin.mpg.de

S2 Text. Simulation of MSE's sensitivity to PSD slope variation.

Our simulations have focused on narrowband rhythmicity as one contributor to time series irregularity. However, MSE is theoretically sensitive to many features that add alter the irregularity of time series, with fixed 1/f slopes. Due to the assumed contribution of variations in autocorrelative structure to signal irregularity, we systematically assessed the impact of variations in pink noise on MSE. For this purpose, we simulated 100 trials of 8 s segments with unit variance and varying pink noise ($\frac{1}{f^x}, x = [.5, 1, 1.2, 1.5]$) as generated using the function f_alpha_gaussian (Stoyanov, Gunzburger, & Burkardt, 2011).

Previous simulations of the impact of varying slopes on 'Original' MSE have produced a multi-scale sensitivity that we consider counterintuitive (e.g., Courtiol et al., 2016; Miskovic, Owens, Kuntzelman, & Gibb, 2016). For white noise signals (x = 0), entropy decreases have been observed towards coarser scales, opposing the notion of 'scale-free' randomness. We and others (Nikulin & Brismar, 2004) argue that this results from increasingly mismatched similarity bounds. Our results closely replicate the traditional observations of scale-dependent entropy crossovers in 'Original' implementations (S7 Figure A), while adding that adequate scale-wise implementation of similarity bounds eliminates such cross-over effects (S7 Figure BC), and instead differentiates different autocorrelative structures by constant offsets in sample entropy (S7 Figure BCD). This result more closely reflects the notion of 'scale-free' irregularity. Notably, a bandpass implementation loses sensitivity to such broadband effects, as narrowband-filtered irregularity is equal across varying slopes (S7 Figure E).

Supplementary References

Courtiol, J., Perdikis, D., Petkoski, S., Muller, V., Huys, R., Sleimen-Malkoun, R., & Jirsa, V. K. (2016). The multiscale entropy: Guidelines for use and interpretation in brain signal analysis. *Journal of Neuroscience Methods, 273*, 175-190. doi:10.1016/j.jneumeth.2016.09.004

Miskovic, V., Owens, M., Kuntzelman, K., & Gibb, B. E. (2016). Charting moment-to-moment brain signal variability from early to late childhood. *Cortex, 83*, 51-61. doi:10.1016/j.cortex.2016.07.006

Nikulin, V. V., & Brismar, T. (2004). Comment on "Multiscale entropy analysis of complex physiologic time series". *Physical Review Letters, 92*(8). doi:10.1103/PhysRevLett.92.089803

Stoyanov, M., Gunzburger, M., & Burkardt, J. (2011). Pink noise, 1/f (alpha) noise, and their effect on solutions of differential equations. *International Journal for Uncertainty Quantification, 1*(3), 257-278. doi:10.1615/Int.J.UncertaintyQuantification.2011003089
