# Supplementary material for: Standard multiscale entropy reflects neural dynamics at mismatched temporal scales: What’s signal irregularity got to do with it?
Source: PLoS Comput Biol. 2020 May 11;16(5):e1007885. doi: 10.1371/journal.pcbi.1007885 (PMC7241858; doi:10.1371/journal.pcbi.1007885)
Supplement: S3 Text — (PDF) [file pcbi.1007885.s003.pdf]

Supplementary Information for

Standard multiscale entropy reflects neural dynamics at mismatched temporal scales

Julian Q. Kosciessa, Niels A. Kloosterman, and Douglas D. Garrett

Email: kosciessa@mpib-berlin.mpg.de; garrett@mpib-berlin.mpg.de

S3 Text. Surrogate analysis of age effects.

The use of multiscale entropy is at least in part motivated by its partial sensitivity to multi-scale, potentially non-linear, signal characteristics, such as phase shifts or cross-frequency coupling. However, the contribution of non-linear characteristics to MSE estimates and modulations thereof is unclear in practice. A principled way to dissociate non-linear signal characteristics from linear signal variance is to use phase-shuffled surrogate data (Garrett, Grandy, & Werkle-Bergner, 2014; Grandy, Garrett, Lindenberger, & Werkle-Bergner, 2013; McIntosh, Kovacevic, & Itier, 2008; Stam, 2005; Takens, 1993; Theiler, Eubank, Longtin, Galdrikian, & Farmer, 1992; Vakorin & McIntosh, 2012).

To probe whether linear contributions were sufficient to explain the main MSE age effects observed in our study, we created surrogate data and estimated 'Original' MSE – including a presumed similarity bound bias – as well as the low-pass variant that matches similarity bounds to the standard deviation of scale-specific signals. In line with previous surrogate analyses for entropy applications (Miskovic, MacDonald, Rhodes, & Cote, 2019), we used an iterated amplitude-adjusted Fourier transform (IAAFT), which minimizes the spurious detection of nonlinearity (Schreiber & Schmitz, 1996). In short, the IAAFT produces surrogate data with random phases, while the power spectrum and value distribution are iteratively approximated to the original data (for an example see S9 Figure A). We separately generated surrogate time series for each subject, channel and pseudo-trial, using a maximum number of 100 iterations until convergence.

Results in S9 Figure show that the surrogate data can recover the main age effects presented in Fig 7 A and C, indicating that linear properties are sufficient to account for the main age effects observed in the original data. This result coheres with a similar surrogate analysis of age effects in resting state data (Courtiol et al., 2016) and suggests at best limited non-linear contributions that were not necessary for the indicated age differences. However, this does not answer the question whether there are also age effects in non-linear contributions after controlling for linear characteristics. To answer this question, we calculated a surrogate ratio score as $\frac{MSE\ (original)}{MSE\ (surrogate)}$, in line with previous surrogate analyses (Miskovic et al., 2019; Schartner et al., 2017). While a score of 1 would indicate the absence of structured information, lower values suggest the presence of non-linear structure in the original data relative to the random structure of surrogates. In contrast with MSE for surrogates or original data only, this ratio indicated similar scale-dependent patterns across 'Original' and low-pass variants in average traces (S9 Figure D, E). At face value, average traces hinted at age-related increases in posterior fine-scale entropy, and age-related decreases in frontal coarse-scale entropy, in line with prior proposals of a shift from global-to-local processing with increased adult age (McIntosh, 2019). However, no significant clusters were indicated via cluster-based permutation tests at traditional thresholds (two-sided p = .025). Even relaxing two-sided significance thresholds to p = .1 only led to the indication of decreased sample entropy with age exclusively at very fine scales and central channels (not shown). We further assessed the correspondence of linear and non-linear effects to the 'Original' MSE age differences in fine- and coarse-scale clusters in the original data. We assessed t-value ratios to evaluate relative effect sizes. We exclusively probed results from the 'Original' implementation given that non-linear results were comparable across implementations. Linear contributions were approximated by t-values for the surrogate data, whereas non-linear contributions were estimated by t-values of the original/surrogate ratio, averaged within the fine- and coarse scale clusters. Linear contributions, approximated by t-values for the surrogate data, accounted for 98% of the original fine-scale effect size and 99% for the coarse-scale effect size. In stark contrast, non-linear contributions captured only .1 % of the original fine-scale effect size, and 20% of the coarse-scale effect size. These results underline that the evaluation of non-linear contributions requires stringent control for linear PSD properties to

evaluate. Smaller (potentially under-powered) non-linear contributions to age effects are further in line with previous surrogate analyses. Crucially, the absence of significant effects suggests that more statistical power is necessary to indicate smaller non-linear effects of interest in future work. Reassuringly, the similarity between surrogate ratio scores for different implementations underline the notion that surrogate analyses provide a powerful tool to identify non-linearities in the presence of linear power differences.

Supplementary References

Courtiol, J., Perdikis, D., Petkoski, S., Muller, V., Huys, R., Sleimen-Malkoun, R., & Jirsa, V. K. (2016). The multiscale entropy: Guidelines for use and interpretation in brain signal analysis. *Journal of Neuroscience Methods, 273*, 175-190. doi:10.1016/j.jneumeth.2016.09.004

Garrett, D. D., Grandy, T. H., & Werkle-Bergner, M. (2014). *The neural forest and the trees: On distinguishing the variance of a brain signal from its information content*. Paper presented at the Annual Alpine Brain Imaging Meeting, Champéry, Switzerland.

Grandy, T. H., Garrett, D. D., Lindenberger, U., & Werkle-Bergner, M. (2013). *Exploring the limits of complexity measures for the analysis of age differences in neural signals*. Paper presented at the Dallas Aging and Cognition Conference, Dallas, TX, USA.

McIntosh, A. R. (2019). Neurocognitive Aging and Brain Signal Complexity: Oxford University Press.

McIntosh, A. R., Kovacevic, N., & Itier, R. J. (2008). Increased Brain Signal Variability Accompanies Lower Behavioral Variability in Development. *Plos Computational Biology, 4*(7). doi:10.1371/journal.pcbi.1000106

Miskovic, V., MacDonald, K. J., Rhodes, L. J., & Cote, K. A. (2019). Changes in EEG multiscale entropy and power-law frequency scaling during the human sleep cycle. *Human Brain Mapping, 40*(2), 538-551. doi:10.1002/hbm.24393

Schartner, M. M., Pigorini, A., Gibbs, S. A., Arnulfo, G., Sarasso, S., Barnett, L., . . . Barrett, A. B. (2017). Global and local complexity of intracranial EEG decreases during NREM sleep. *Neurosci Conscious, 2017*(1), niw022. doi:10.1093/nc/niw022

Schreiber, T., & Schmitz, A. (1996). Improved surrogate data for nonlinearity tests. *Physical Review Letters, 77*(4), 635-638. doi:DOI 10.1103/PhysRevLett.77.635

Stam, C. J. (2005). Nonlinear dynamical analysis of EEG and MEG: review of an emerging field. *Clinical Neurophysiology, 116*(10), 2266-2301. doi:10.1016/j.clinph.2005.06.011

Takens, F. (1993). Detecting Nonlinearities in Stationary Time Series. *International Journal of Bifurcation and Chaos, 3*(2), 241-256. doi:10.1142/S0218127493000192

Theiler, J., Eubank, S., Longtin, A., Galdrikian, B., & Farmer, J. D. (1992). Testing for Nonlinearity in Time-Series - the Method of Surrogate Data. *Physica D-Nonlinear Phenomena, 58*(1-4), 77-94. doi:10.1016/0167-2789(92)90102-S

Vakorin, V. A., & McIntosh, A. R. (2012). Mapping the Multiscale Information Content of Complex Brain Signals. *Principles of Brain Dynamics: Global State Interactions*, 183-208.
