# Supplementary figures and images for: Standard multiscale entropy reflects neural dynamics at mismatched temporal scales: What’s signal irregularity got to do with it?
Source: PLoS Comput Biol. 2020 May 11;16(5):e1007885. doi: 10.1371/journal.pcbi.1007885 (PMC7241858; doi:10.1371/journal.pcbi.1007885)

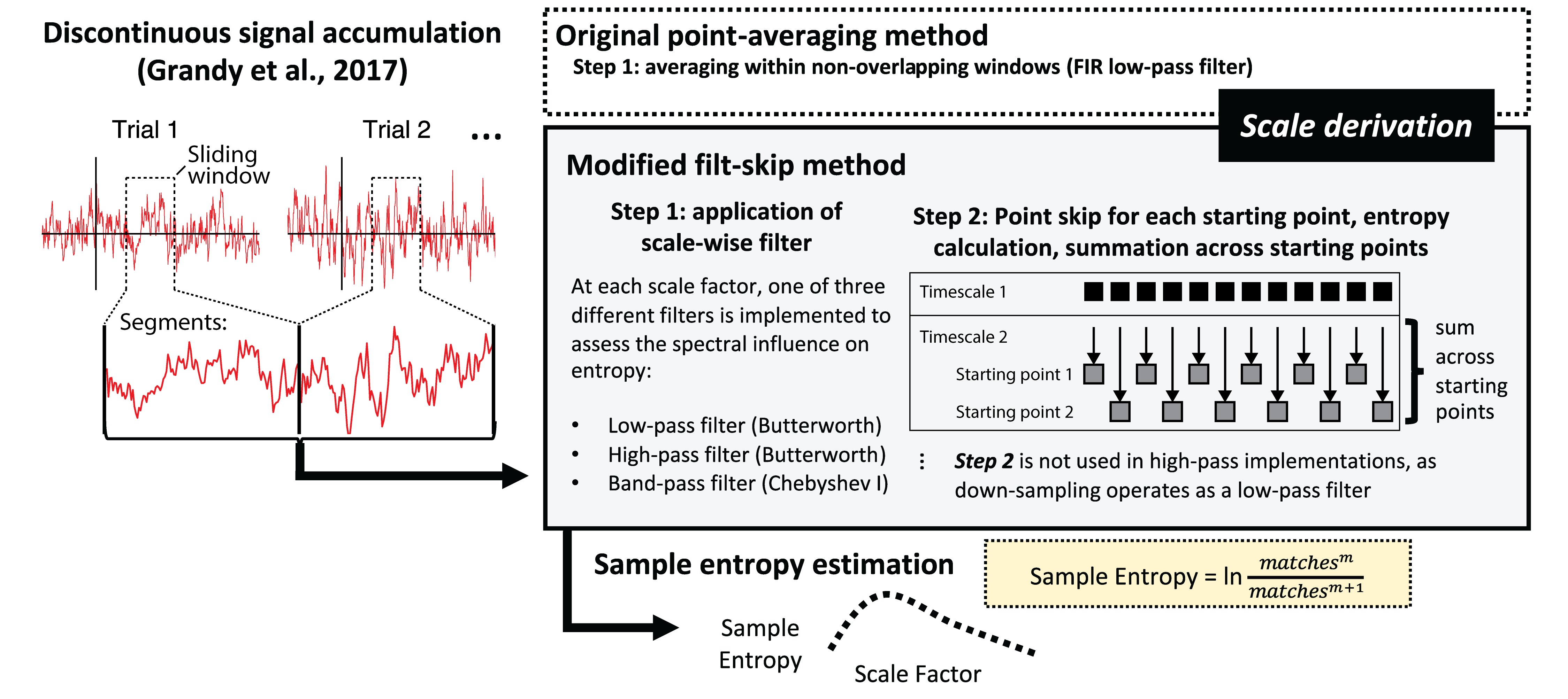

Supplement: S1 Fig — First, mMSE uses data aggregation across (here: pseudo-) trials to allow the estimation of coarse scales also from sparse neuroimaging data [64]. These aggregated signals are then filtered at each scale prior to sample entropy calculation. The ‘Original’ implementation uses ‘point averaging’ for different scale factors, which is equivalent to a FIR low-pass filter. In adapted applications, we used a two-step implementation, which we refer to as ‘filt-skip’, which first applies a scale-wise low-, high- or band-pass filter, and then performs point skipping to down-sample the resulting signals. Finally, the sample entropy of these signals is similarly assessed using the sample entropy algorithm, which results in multiscale entropy estimates. Figure adapted with permission from [121]. (TIF) [file pcbi.1007885.s004.tif]

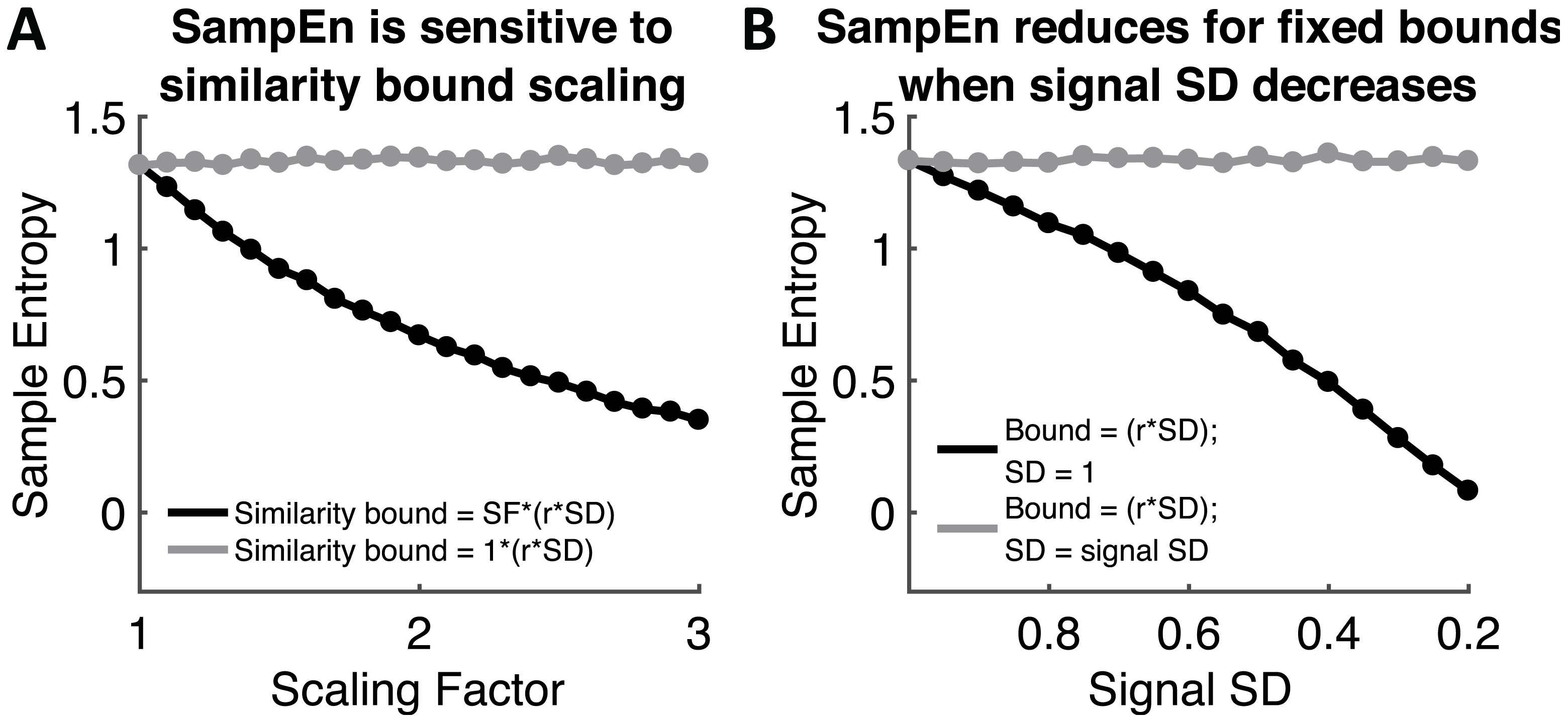

Supplement: S2 Fig — (A) The plot shows the sample entropy of simulated white noise signals with constant signal standard deviation (SD) of 1, but varying similarity bounds. We denote this as a function of a scaling factor (SF) to highlight that such variation may arise from either variation in r, SD or both. Note that the r parameter is usually fixed and the SD matches the signal SD (gray line), thus normalizing total signal variance. However, when the similarity bound systematically increases relative to the signal SD, entropy estimates progressively decrease (black line). (B) A similar scenario applies when fixed and large bounds are applied to signals of decreasing variance, as is the case across MSE time scales due to scale-wise filtering (Fig 2). Whereas no bias is observed when scale-wise signal SD is used for the calculation of similarity bounds (grey line), entropy estimates systematically decrease when the SD of the original signal are used (black line). Hence, the mismatched similarity bounds introduced entropy decreases although no changes to the structure of the (here white noise) signals were introduced. (TIF) [file pcbi.1007885.s005.tif]

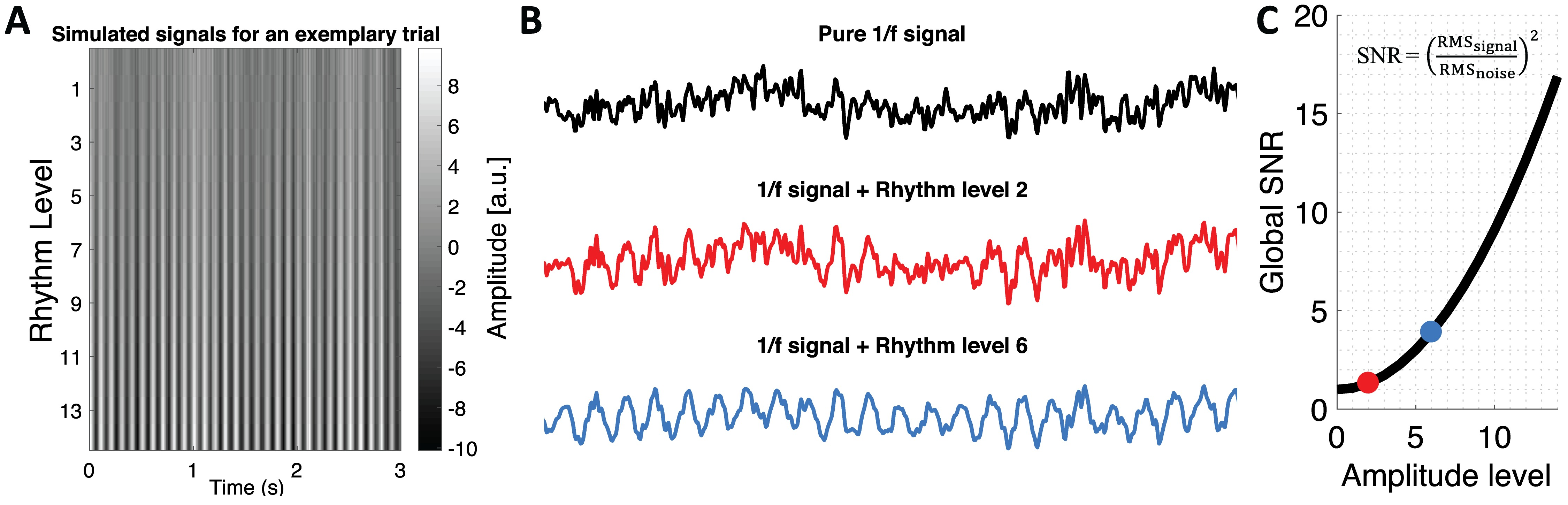

Supplement: S3 Fig — (A) Top-down view of time-series from an exemplary simulated trial for a pure 1/f signal pink noise signal and at different magnitudes of added alpha rhythmicity. (B) Exemplary time series in 2D view. The red time series indicates an example time series for the level of rhythmicity shown in Fig 5. (C) Simulated SNR as a function of amplitude level. The dots indicate SNR for the levels depicted in panel B. (TIF) [file pcbi.1007885.s006.tif]

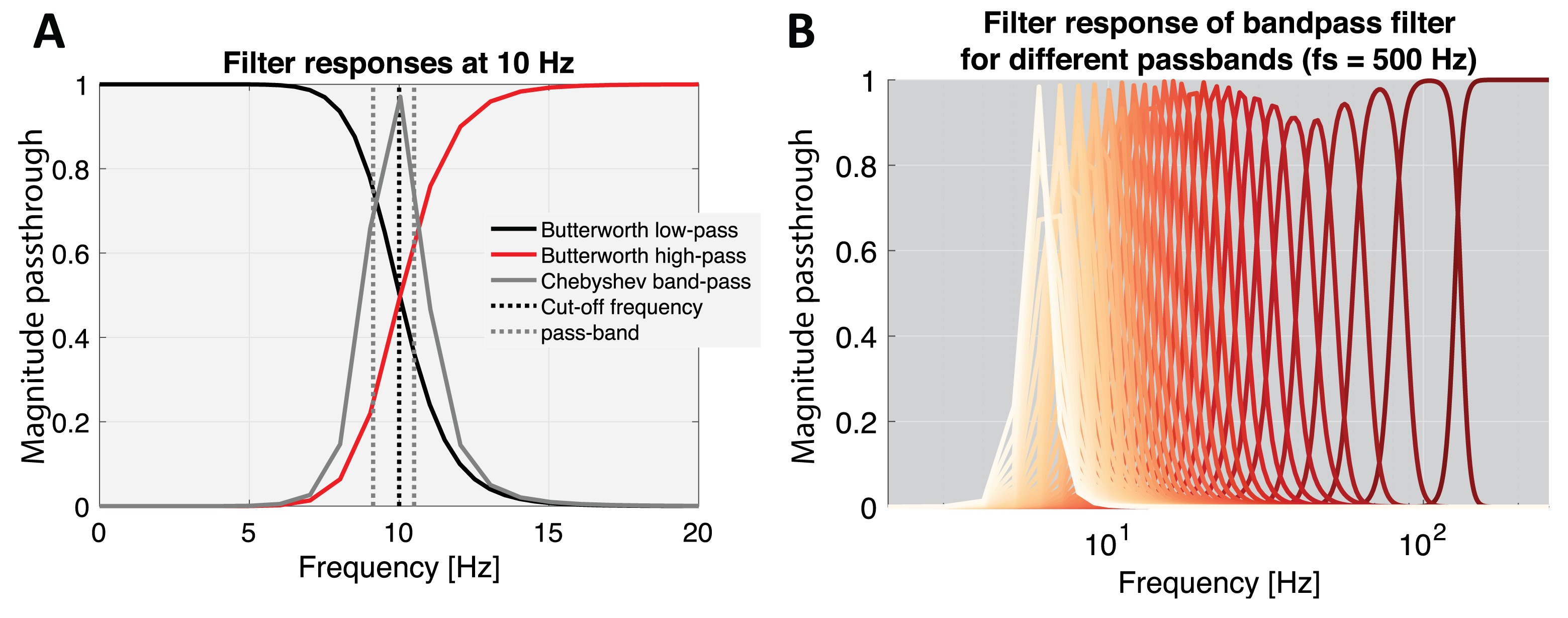

Supplement: S4 Fig — (A) Filter magnitude responses at 10 Hz. Note that magnitude responses have been squared due to two-pass filtering to achieve zero-phase offsets. (B) Filter magnitudes of Bandpass filters (3rd order type I Chebyshev filter with 1dB passband ripple) at different time scales (red-to-orange indicating fine-to-coarse time scales). Note that only a high-pass filter (6th order Butterworth filter) is applied at the first scale. (TIF) [file pcbi.1007885.s007.tif]

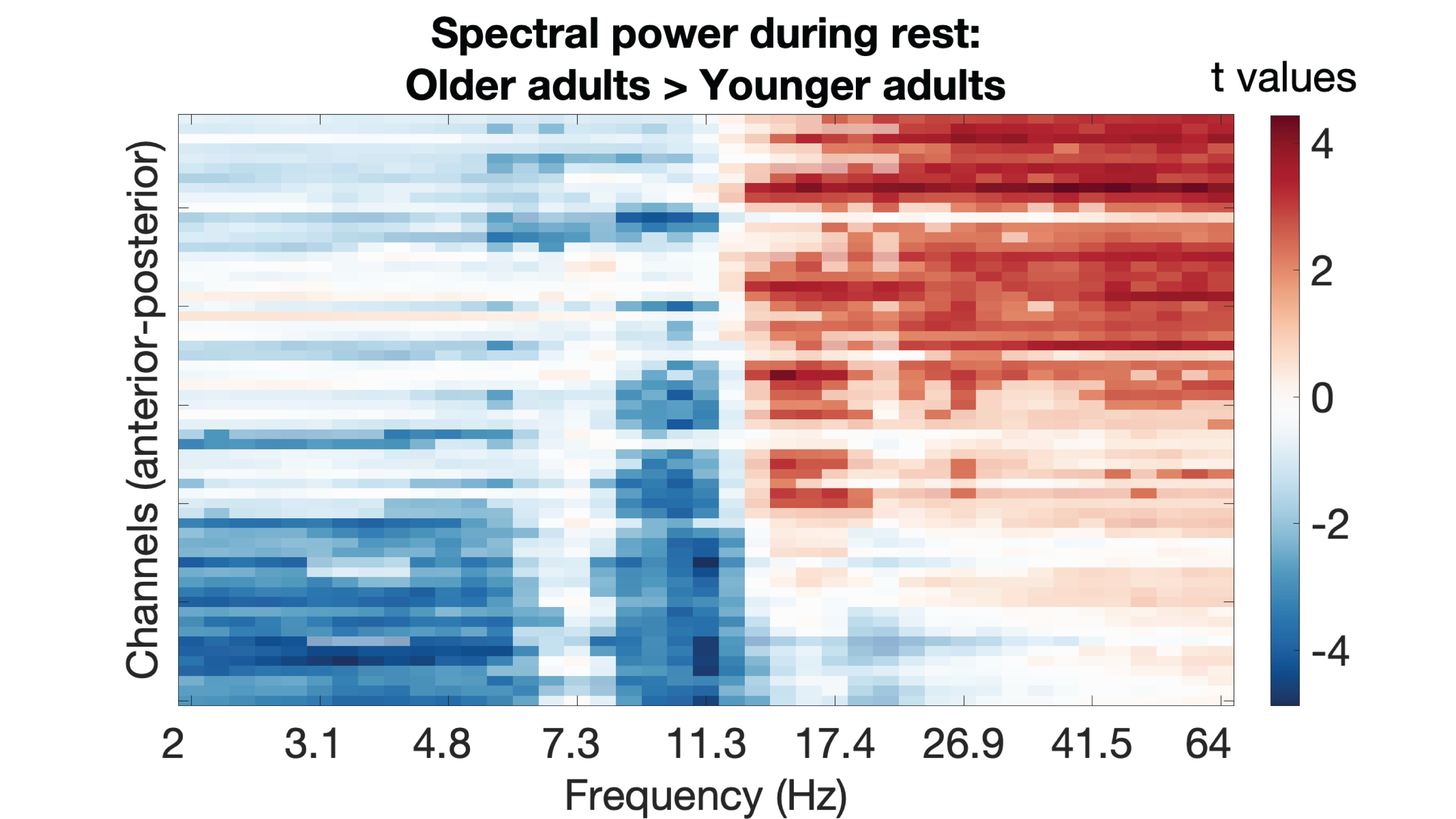

Supplement: S5 Fig — Statistical significance (p < .05) was assessed by means of cluster-based permutation tests and is indicated via opacity. (TIF) [file pcbi.1007885.s008.tif]

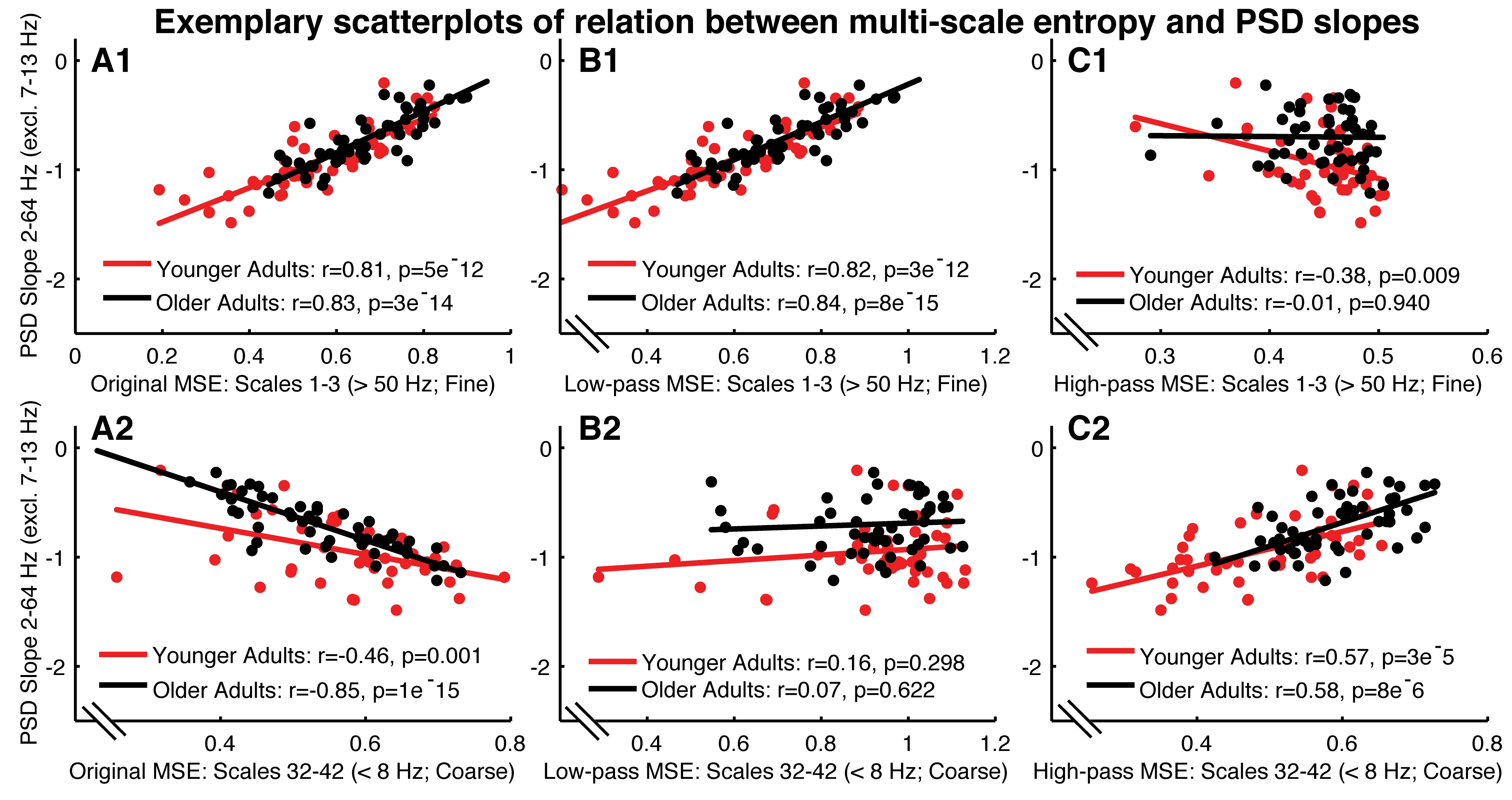

Supplement: S6 Fig — ‘Original’ settings indicate a strong positive association at fine scales (A1) that turns negative at coarse scales (A2), likely due to coarse-scale biases by the scale-invariant similarity criterion. In line with this notion, scale-wise adaptation of thresholds retains the fine-scale effect (B1), while abolishing the coarse-scale inversion (B2). Crucially, the entropy of exclusively high-frequency signals does not positively relate to PSD slopes (C1), whereas the association reemerges once slow fluctuations are added into the signal (C2). (TIF) [file pcbi.1007885.s009.tif]

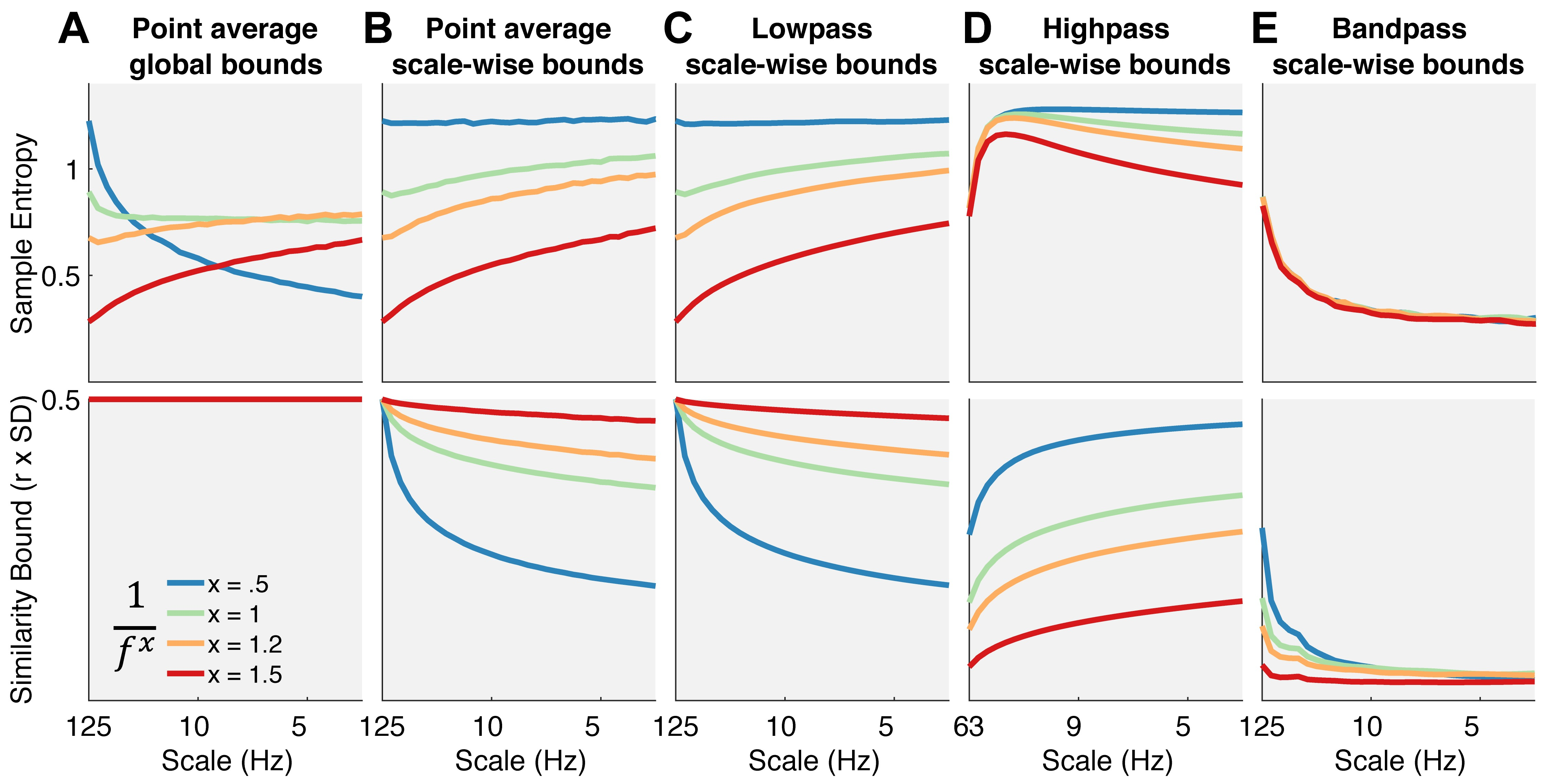

Supplement: S7 Fig — (A) Using traditional implementations, 1/f variation introduces scale-dependent crossover effects, including scale-dependent entropy decreases for the signals approaching white noise. (B, C, D) In contrast, control for scale-wise variance indicates broad scale entropy offsets without crossovers. (E) Bandpass entropy is not modulated by broadband effects, as expected by the absence of multi-scale information at local scales. (TIF) [file pcbi.1007885.s010.tif]

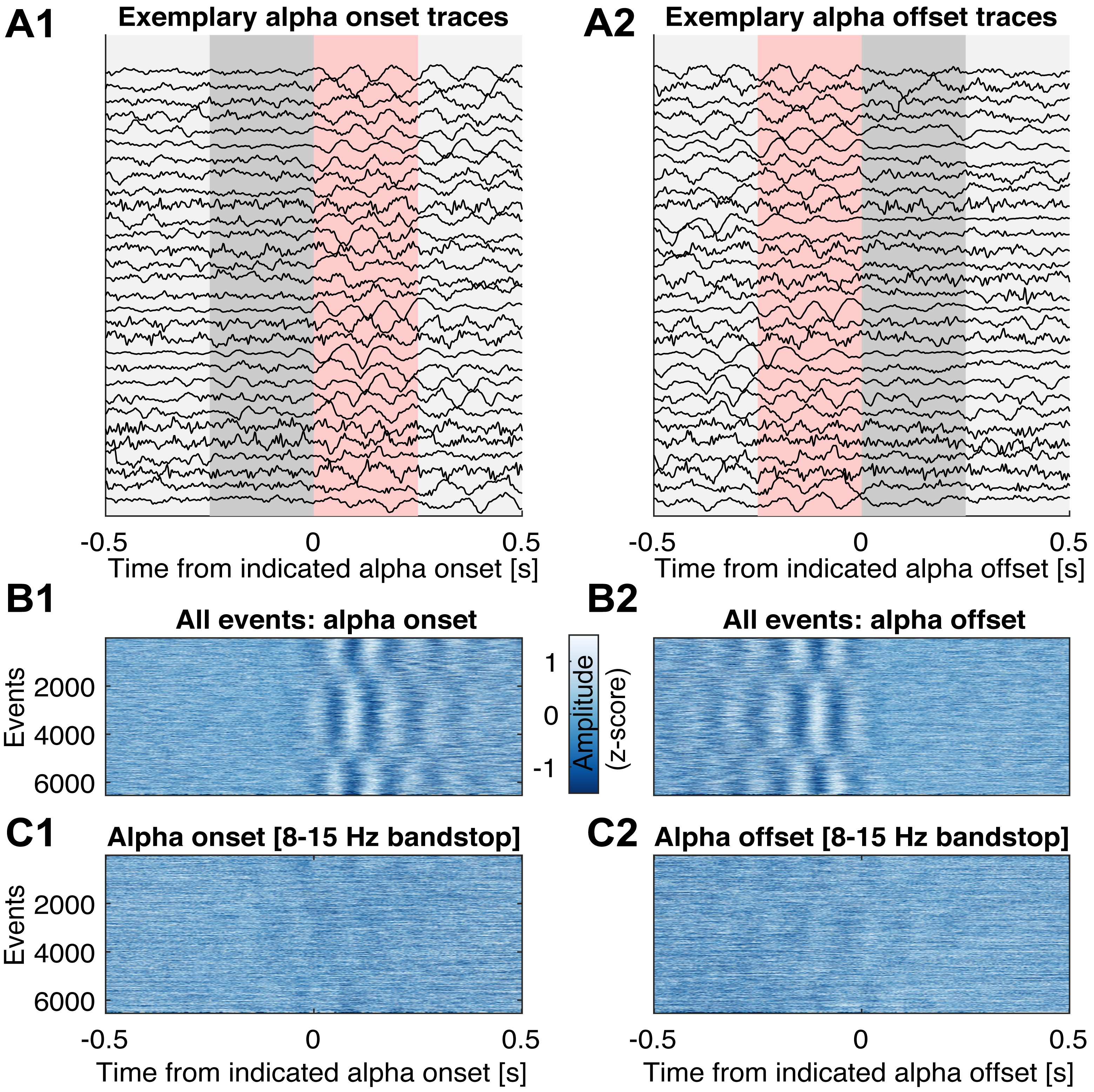

Supplement: S8 Fig — (A) Thirty randomly selected traces across subjects for alpha on- (A1) and offsets (A2). The grey background indicates the 250 ms pre- and post-alpha windows used for the calculation of sample entropy (see Fig 11). The red background highlights segments following indicated alpha onsets, and preceding alpha offsets, that were used to assess irregularity during transient alpha events. Note that 250 ms segments may overlap in the case of short rhythmicity of around 3 cycles. (B) All events around on- and offsets. Data were sorted by the instantaneous phase at +100 ms after indicated alpha onset (B1) and -100 ms prior to indicated alpha offset (B2). Instantaneous phase was calculated from a Hilbert transform applied to 8–15 Hz bandpass filtered signals. (C) Same as in B, but plotted for signals after 8–15 Hz bandstop filter application. All displayed traces were z-scored for presentation purposes. (TIF) [file pcbi.1007885.s011.tif]

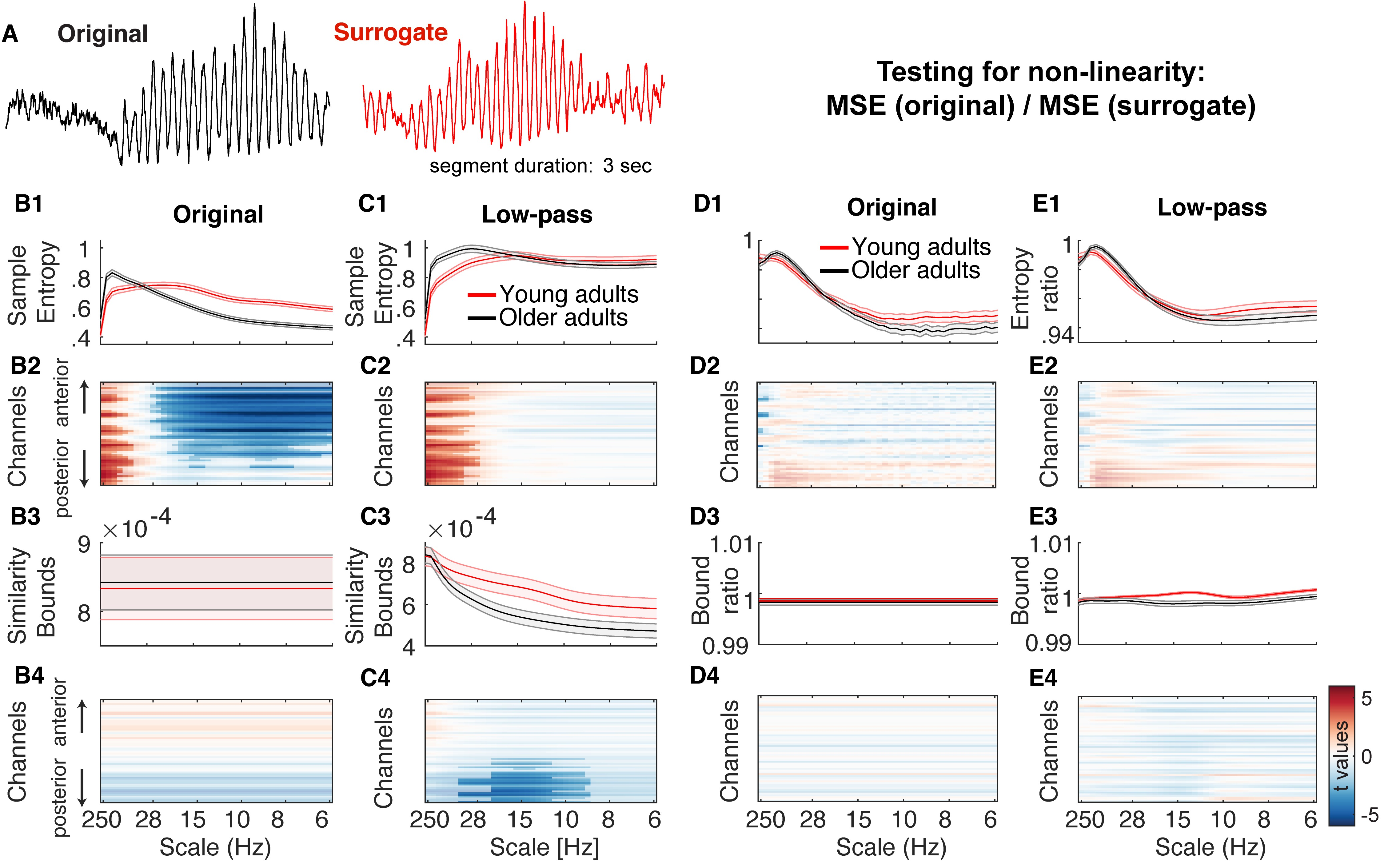

Supplement: S9 Fig — (A) Examples of original and surrogate data for a random 3 s segment from an occipital channel with strong alpha rhythms. Phase randomization alters higher-order (non-linear) frequency interactions while preserving the linear power characteristics of the original data. If non-linear contributions are necessary for MSE age effects, no age effects should be indicated for entropy estimates of surrogate data (B) Results for “Original” MSE analysis on phase-shuffled data indicate similar effects as observed for original data (Fig 7A), suggesting that linear characteristics were sufficient for the observed age effects. (C) Results for low-pass MSE analysis on phase-shuffled data indicate similar effects as observed for original data (Fig 7C), suggesting that linear characteristics were sufficient for the observed age effects. (D, E) In addition to assessing the necessity of non-linear contributions, we further assessed whether age differences would be indicated for non-linear contributions, after accounting for linear power characteristics. The ratio of MSE estimates for original vs. surrogate data indicates unique non-linear contributions for either age group. The obtained results were remarkably similar for both original (D) and low-pass implementations (E), indicating the successful elimination of power-based biases. However, no statistically significant age differences were indicated, suggesting that non-linear contributions are at most minor, and may require higher statistical power for their assessment. (TIF) [file pcbi.1007885.s012.tif]
