# Supplementary material for: Standard multiscale entropy reflects neural dynamics at mismatched temporal scales: What’s signal irregularity got to do with it?
Source: PLoS Comput Biol. 2020 May 11;16(5):e1007885. doi: 10.1371/journal.pcbi.1007885 (PMC7241858; doi:10.1371/journal.pcbi.1007885)
Supplement: Attachment — Submitted filename: RhythmsEntropy_PCB_v2_Reviewer Letter.pdf [file pcbi.1007885.s013.pdf]

Letter to the reviewers

Dear reviewers,

We thank you for your thorough review of our initial submission and your constructive comments. We were encouraged by your positive feedback and hope that we have satisfactorily addressed your remaining questions and comments with our revision. In the following sections, we address each of those comments and highlighted changes in the revised version of the manuscript, alongside marking changes in the main text in **blue**.

With our revision, we attend to the request to "show an actual relevance in terms of better understanding of the biological mechanisms behind the signal properties". For this, we emphasized the present ambiguity of interpretations given biases in the field and extended the discussion of observed age-related changes. Crucially, we added a novel analysis that leverages the detection of rhythmic episodes at the single-trial level to assess non-trivial associations between transient alpha rhythms and broadband sample entropy. We believe that this presents a significant use case for neurobiological insights, while simultaneously underlining the core message of our manuscript that a differentiation between narrow- and broadband activity is crucial for such insights.

For ease of communication, we repeat the reviewer's full comments in boxes, and repeat specific sections in red if those comments contained multiple specific points. We repeat relevant sections from our main text in blue.

Reviewer #1:

This technical paper investigates thoroughly, in simulations and with representative EEG data, two issue that affect the interpretability of multiscale entropy (MSE), a widely used tool for the quantification of the complexity of physiological time series performed at multiple time scales: the impact of one specific parameter (the "similarity bound") on the assessment of complexity at long (coarse) time scales, and the impact of dominant low frequency oscillations on the assessment of complexity at short (fine) time scales.

The two issues are actually well known in the literature, and (as also partially acknowledged by the authors) corrective methodological approaches have been proposed to successfully deal with them (see specific comments below). This aspect should be better emphasized in the paper. Nevertheless, in spite of the limited methodological novelty and neurophysiological appeal of the results, the messages conveyed by the paper are important, because it is true that MSE is often misused or at least used not appropriately, and that inferences based on this measure must be made with full awareness of its meaning and limitations (which is often not the case in neuroscience and physiology applications). Therefore, I recommend acceptance of this paper after the content is simplified, in order to focus on the specific message, some interpretations are better placed in the context of the existing methodological literature, and the general claims are smoothed a bit, in order to better acknowledge that existing methodological solutions already make MSE a useful tool - provided that it is used consciously.

We thank the reviewer for their constructive review. We agree with the reviewer that the problems we highlight have been noted before, and have stated accordingly in our manuscript (specifically relating to Issue 1): "This issue has been recognized previously (Nikulin & Brismar, 2004), and provided a rationale for recomputing the *similarity bound* for each time

scale (Faes, Porta, Javorka, & Nollo, 2017; Nikulin & Brismar, 2004; Valencia et al., 2009; Xiong, Faes, & Ivanov, 2017). But despite the benefits of this refinement that was already proposed fifteen years ago, our review of the literature revealed that the use of global bounds remains dominant in over 90% of neuroscientific MSE applications (see S1 File) and in previous validation work (Courtiol et al., 2016)." (lines 151ff.) However, in light of neuroscientific applications, we highlight that proposed solutions to these issues are not currently applied, leading us to draw the conclusion that these issues (and particularly their practical consequences) are not sufficiently understood to motivate the adoption of techniques that try to deal with them. We believe that this is in large part due to ambiguous associations between spectral power and MSE. This ambiguity becomes clear also when reading prior 'interpretation guides to MSE' in neuroscientific applications (Courtiol et al., 2016).

We completely agree with the reviewer (and others) that MSE remains a useful tool, but only once its limitations are known, and attempts are made to overcome them. We hope to have clarified this point in our revision (see particularly *Recommendations for future applications*).

We respectfully disagree with the reviewer's assessment of low methodological novelty or neurophysiological appeal. First, while refined versions attempt to deal especially with issue 1 (i.e., global similarity bounds) (Faes et al., 2017; Nikulin & Brismar, 2004; Valencia et al., 2009; Xiong et al., 2017), we consider issue 2 (i.e., contribution of slow and fast contributions to fine scales) to be largely unresolved – and sparsely discussed. We are not aware of attempts to employ high-pass, band-pass or band-stop filters to derive scale-wise estimates, despite no clear argument why low-pass filters provide an optimal approach for scale-wise entropy calculations. Second, we highlight first evidence that narrowband entropy implementations may be particularly sensitive to non-stationarities in rhythmic engagement, using recent advances in rhythm detection (*Narrowband MSE indicates age differences in signal irregularity in alpha and beta band*). Finally, we have added an analysis that adds neurophysiological appeal to our results (*Rhythmic alpha events transiently reduce broadband signal irregularity*), while clarifying a core methodological issue concerning circularity, which becomes important in questions concerning the coupling of entropy to specific neurophysiological features. We hope that this convinces the reviewer beyond the original communicative appeal of the manuscript.

Specific:

- The effect of the signal variance (here reflected in the term "similarity bound") on the original formulation of MSE is long known (e.g., from Valencia et al. [R1]). Here it is confirmed in simulations, and its effects are highlighted in the practical application studying age differences in resting state EEG. The proposed solution to compute sample entropy based on the variance of the signal after the change of scale (here denoted as "use of local similarity bound") is also well established (it is for example used in linear parametric estimators of MSE (e.g., Faes et al. [R2])). The recommendation to abandon the original MSE formulation (line 678) is valid, but should be substantiated more clearly in terms of existing modifications of the MSE algorithm (like the refined MSE).

We thank the reviewer for bringing the work by Faes et al. (2019); Faes et al. (2017) to our attention. We were happy to include this highly relevant work in our discussion (see lines 170ff., 786ff., 958ff., 974ff.) to better contextualize our work within the existing literature and were happy to read that Faes et al. have provided clear discussions of this issue in their own work. We hope that this clarifies that methods to overcome these biases exist, but would also like to highlight that our previous submission clearly stated the fact that this issue has been

known for some time, and has been dealt with in the methodological literature. Crucially however, the fact remains that these advances are overlooked by the vast majority of published MSE papers (as suggested by our literature review). To us, this means that neither typical reviewers nor editors of MSE papers are aware of how bad these issues can become. We are moreover not aware of evidence that clearly highlights the arising challenges to practical inference. We remain convinced that our detailed example of the bias and its consequences will contribute to the adoption of 'refined' approaches for future applications, which we consider the minimum requirement for novel neurocomputational insights. We have extended the discussion of this conclusion in the revision (see lines 786ff., 958ff., 974ff.).

Also the diffuse reflection of slow oscillations on entropy estimated even at fine time scales (i.e., a contribution of low frequencies to broadband signals) is expected, since at fine time scale the signal contains both low and high frequencies which can both contribute to the irregularity. In particular, the dominance of very low frequency components (where 'very low' relates to the sampling frequency and the length of the time series) introduces trends in the analyzed signal which can be assimilated to nonstationary behaviors; this precludes a proper evaluation of Sample Entropy, because stationarity is a formal prerequisite to the evaluation of entropy measures. This issue is thoroughly dealt with in recent works (e.g., Xiong et al. [R3]). Solutions have been proposed, either based on simple detrend of the time series via highpass filters in order to allow MSE to focus on short-term dynamics without the biasing effect of slow trends, or based on incorporating the long-range correlations into parametric models for the estimation of MSE (Faes et al. [R4]) in order to describe both long- and short-term dynamics in MSE analysis. These aspects should be better emphasized in the paper, in order both to better place the work in the context of the existing literature and to substantiate the recommendation of using spectral filters to deal with the second issue highlighted in this work.

Again, we thank the reviewer for the additional references which we were not aware of – and have not been employed in neuroscientific applications so far. We happily included them where appropriate (see lines 786ff., 958ff., 974ff.).

We agree with the reviewer that the diffuse reflection of broadband content is a straightforward characteristic at early scales. However, this does not help the 'intuitive' interpretation of a scale. It is ambiguous, whether, fine-scale effects are most strongly related to broadband or narrowband phenomena, and within the latter low- or high-frequency contributions. Our analyses suggest that indeed both low- and high-frequency contributions are necessary for links to spectral slopes to emerge. We consider this result non-trivial, as high collinearity between high-frequency fluctuations and spectral slopes would indicate that high frequencies suffice for this link to emerge.

Moreover, this is not limited to autocorrelation. We focus on rhythmic events as a principle descriptor of time scales, and propose that either bandpass or bandstop filters may be optimal to identify the necessity and sufficiency of narrowband contributions to effects of interest. This generalizes the idea of using "highpass filters in order to allow MSE to focus on short-term dynamics without the biasing effect of slow trends".

The paper correctly questions the interpretation often given to MSE that it reflects the complexity of the analyzed signal observed at specific time scales. In doing this, however, it should be stated more explicitly that the mismatch between specific temporal scales and MSE values is a defining feature of this measure, which distinguishes it from simple spectral analysis. The use of lowpass filters encompassing several temporal scales is indeed important

in order to allow the measure to capture complex (possibly nonlinear) behaviors like the superposition of rhythms and at different frequency and their weaker or stronger interaction. Therefore, it should emerge more clearly from the paper that, while it is incorrect to interpret MSE as a scale-specific measure, the "scale" focused by this measure is actually an upper limit of the range of scales analyzed (i.e., at "scale 1", all signal frequencies are considered, and at scale tau all frequencies lower than fs/(2+tau) are considered – where fs is the sampling frequency).

We thank the reviewer for highlighting that this was not clear from the previous draft. We generally agree with the reviewer on the raised points and hope to have addressed these issues with additions to the discussion.

However, we would like to address the specific point raised: "The use of lowpass filters encompassing several temporal scales is indeed important in order to allow the measure to capture complex (possibly nonlinear) behaviors like the superposition of rhythms and at different frequency and their weaker or stronger interaction."

While this is true, we believe it overstates the importance of low-pass filtering for scale-wise entropy estimates. We do not perceive principled arguments against high-pass filters (as noted above by the Reviewer and used in our manuscript), or narrow band-stop filters, as used in our added analysis. Both of these implementations preserve multi-scale information, but shift the focus away from higher specificity to slow fluctuations toward either higher specificity to fast fluctuations or the principled exclusion of narrowband signals of no interest. In addition, we do not share the opinion that a multi-scale sensitivity is the only (or defining) property that differentiates the MSE measure from spectral analysis. Estimates are intended to become more specific to low-frequency content with increasing scale after all. As such, increasing loss of multi-scale sensitivity (and increasing specificity towards slower fluctuations) is an inherent property of the MSE algorithm that distinguishes it from simple sample entropy. We hope that the added results regarding narrowband alpha fluctuations *(Rhythmic alpha events transiently reduce broadband signal irregularity)*, as well as the added discussion (see next response) are convincing and clarify this point.

Related to the comment above, two issues should be also considered:
(i) not only it is wrong to assume that MSE reflects a specific time scale, but also it makes little sense to look scale-specific MSE values through narrowband filters; the authors correctly show that narrowband filters reconcile MSE with the spectral content (e.g. in Fig. 4E), but they should acknowledge that in that case MSE itself becomes of little use, since it merely reflects the presence of specific oscillations and thus it adds little or no information to much simpler spectral analysis (the evaluation of nonlinear effects and cross-frequency interactions is precluded by the narrowband filter).

We agree with the reviewer that a narrow bandpass (but not bandstop) approach challenges the detection of multi-scale phenomena. However, we regard the situation as more complex, given that features of irregularity can also be scale-specific. We have added a discussion of this point, which we replicate below.

"Notably, a narrowband approach may warrant different use cases than broadband entropy. In particular, the sensitivity to multi-scale information, such as cross-frequency interactions and waveform shape, is a defining characteristic of (and motivation for using) entropy as opposed to spectral analysis. However, this sensitivity trades off with specificity when a narrowband approach is chosen, which by definition enforces a more rhythmic appearance than the raw

signal may convey [106]. Nonetheless, frequency-specific phenomena such as variations in the amplitude or the presence of rhythmic events are complementary signatures of irregularity in their own right. For example, long-range temporal correlations (LRTCs) of narrowband amplitudes provide an alternative window on the irregularity of temporal dynamics [107-109]. As such, targeted filter applications – either chosen *a priori* or as a follow-up to broadband entropy effects – may prove useful to delineate spectrally specific effects at directly interpretable neural time scales. Hence, we do not regard narrowband MSE as a replacement for the traditional low-pass implementation of MSE, but rather as a parallel tool for the exploration and probing of broadband effects. Moreover, sensitivity to broad-scale phenomena remains high when band-stop filters are used (e.g., Fig 11), highlighting the general feasibility of applying narrowband filters to derive broadband insights beyond the band-stop range." (lines 849ff.)

(ii) the message that MSE is of little use because it reflects spectral content at mismatched temporal scales (given first of all in the title of the article) is misleading and should be tuned down. While it is true that the balance among spectral content at different frequencies has an effect on the MSE value, such a balance is one of the factors that determines signal complexity (e.g., white noise is highly complex, an oscillation embedded in noise is less complex, the presence of another oscillation at different frequency increases complexity, the complexity increases with the bandwidth of stochastic oscillations, and so on)

We have not stated that MSE is of little use and hope that this is not the perceived message from our work. Instead, we hope to offer a constructive critique and highlight specific controls that "may go a long way toward establishing unique, complementary, and valid contributions of MSE in future work" (lines 918f.). Moreover, our conclusions summarize our perspective that "[…] MSE effects may be taken as a starting point to explore the linear and nonlinear features of brain signals (e.g., Simpraga et al., 2017). We believe that empirical identification of the unique predictive utility of MSE will advance the quest for reliable mechanistic indicators of flexible brain function across the lifespan, and in relation to cognition, health, and disease." (lines 934ff.)

The title is not meant as a discouragement of MSE analyses, but as a (admittedly ambiguous) question concerning how the timescale of neural dynamics (as indicated via spectral power) and MSE are related. To reduce the impression that we are dominantly arguing for a spectral perspective – a concern shared by Reviewer 3 – we have altered the title to "Standard multiscale entropy reflects neural dynamics at mismatched temporal scales: What's signal irregularity got to do with it?". This retains the focus of the original title on investigating the relationship between the timescale of neural dynamics to MSE (and empirical mismatches therein), while highlighting that we regard spectral power merely as a principled reflection of the timescale of neural dynamics (or the lack of a dominant time scale in the case of scale-free broadband contributions).

We agree with the reviewer on the expected relation between PSD slopes and fine-scale entropy and would like to highlight the related statement from the manuscript: "More generally, the association between broadband signal entropy and spectral slopes coheres with the notion that shallower slopes have a more 'noisy' or irregular appearance in the time domain. Thus, spectral slopes and temporal predictability are – at least in part – different perspectives on the same signal characteristic." (lines 753ff.) This comment appeared in a slightly altered form in the previous submission.

We agree with the reviewer that 'irregularity' is a multi-faceted concept, and highlighting biases related to it, as well as identifying clear features, aids in clarifying use cases and interpretations. We now emphasize the fact that further work with reduced biases is necessary to highlight the complementary benefits of MSE estimation, especially at coarse scales.

The paper is very well written, but it is likely too long compared to the message that is given. An effort to focus on the main message would be helpful to better convey it.

We thank the reviewer for indicating a clear structure. We believe that other's attempts at conveying these issues have presented them in either too-dense mathematical detail or in so brief a manner that these important insights were not tractable for use in neuroscientific applications.

We also consider the following statement by reviewer 3 relevant for this point: "I was very impressed by […] the clear demonstration of how these methodological points work, and what they mean." We feel our work represents a sweet-spot in the available literature on issues with/interpretations of MSE. We argue that a clear communication of the methodological issues is more important than brevity in this case, esp. for neuroscientists that may generally not be aware of MSE's technical details, but may be interested in its applications and the interpretation of past results due to conceptual overlap. Complementary to our more in-depth sections, we hope that our main messages are succinctly communicated in our recommendation section, and are happy to see that all of the reviewers were able to concisely summarize our conclusions.

[R1] Valencia, J. F., et al. (2009). Refined multiscale entropy: Application to 24-h holter recordings of heart period variability in healthy and aortic stenosis subjects. IEEE Transactions on Biomedical Engineering, 56(9), 2202-2213.

[R2] Faes, L., et al. (2017). Efficient computation of multiscale entropy over short biomedical time series based on linear state-space models. Complexity, 1768264.

[R3] Xiong, W., et al. (2017). Entropy measures, entropy estimators, and their performance in quantifying complex dynamics: Effects of artifacts, nonstationarity, and long-range correlations. Physical Review E, 95(6), 062114.

[R4] Faes, L., et al. (2019). Multiscale information storage of linear long-range correlated stochastic processes. Physical Review E, 99(3), 032115.

Reviewer #2:

This manuscript, by Kosciessa and colleagues, investigates methodological properties of applying multiscale entropy (MSE) to neural field data, such as EEG. The research question is broadly, whether MSE actually measures signal irregularity across scales, in the way that it is typically interpreted to do. More specifically, this investigation centers on two key methodological points about how typical approaches compute and analyze the 'scales' that are foundation of the 'multi-scale' approach, specifically:
whether using a global similarity bound biases measures across scales, since the coarse-graining (or analogous) procedure reduces the variance as one restricts the data to different scales, and so a globally computed similarity bound effects each scale differently and thus conflates signal regularity and signal variance; and
whether the coarse graining procedure is specific to presumed scales, or whether, for example,

scales that include, but are not limited to, high frequency activity capture entropy measures that are specific to high frequency activity, or whether the low frequency activity also impacts the measures and thus conflates the typical interpretations of measures at particular scales.

The authors demonstrate, in a series of simulations, that these two methodological issues are indeed problems, such that global similarity bounds and broadband data lead to biases that violate how the resulting measures are typically interpreted. In analyses of EEG data, they further demonstrate how this is the case, showing how patterns of MSE relate to different similarity bounds and coarse graining approaches. These analyses also demonstrate how MSE measures relate to power. Through this work, they propose and investigate solutions to these problems: notably to recompute similarity bounds specific to the variance at each scale, and to use filters to isolate data more specifically to desired scales.

Overall, I commend the authors on a rigorous and methodologically minded paper, that demonstrates methodological insight into a relatively common method in the field, demonstrated through what I find to be a compelling and well chosen empirical analysis. I was very impressed by the methodological rigour of the work, the open nature of the code and data, and the clear demonstration of how these methodological points work, and what they mean. This seems to me to be important work for the field, and impactful work in terms of thinking of how to interpret many studies that have employed these methods.

Overall, I did not find any major issues with the overall framing, logic or main results of this paper. I also find it to be well written. My review is therefore focused on a what I consider to be some minor revisions that include some clarifications regarding their proposed adaptations MSE, a possible small extension to the simulations, and some possible tweaks to the figures.

We thank the reviewer for their concise summary of our work and for assigning high value to it.

Comments:

1) Bandpass Filtering

My main conceptual question / comment is about the use, interpretation and recommendation for the bandpass filtered version of the entropy. I like the idea of this approach, and the justification of how it addresses the issues raised, and is a design that actually specifies scales precisely. However, it still seems slightly unclear what it captures, and if (or to what extent) this is a) the same as the typical conceptualization of MSE and b) distinct from other measures (such as power and burst analysis).

In particular, the demonstration that alpha and beta differ in bandpass MSE between groups is very interesting. However, given that the alpha does relate to power, and the beta does relate to the burst analysis, I found it unclear if the there is a conclusion as to whether this MSE approach adds information over and above a combined power & burst analysis of rhythmic activity. The implication in the results appears to be that the difference in beta is proposed to be related to different bursting statistics of rhythmic activity between groups. If this is the appropriate interpretation, it seems to me that a) it is not entirely clear to me that tracking burst properties (such as probably and duration of bursts) of rhythmic activity is quite the same idea as MSE has been proposed to relate to - of signal irregularity (seemingly, more broadly). This could perhaps just be a conceptual issue, as burst dynamics are clearly an aspect of signal (ir-)regularity, but my impression is that entropy measures are more typically

thought of as representing the 'eracticness' of time series, and not necessarily the propensity for bursts of rhythmicity. It is also somewhat unclear to me if this MSE analysis offers something more than could be gleaned from a power and burst analysis of these bands.

Specifically, I would appreciate a comment in the manuscript that addresses 1) whether this MSE measure adds something more than would be gleaned from a power and burst analysis and 2) whether, if one does try this, the results should be interpreted somewhat differently than how 'original MSE' has typically been interpreted and discussed.

We thank the Reviewer for this clear comment, which mirrors a concern from Reviewer 1.

Indeed, we interpret our results as a reflection of ‘burst’ statistics in the present case, which in and of itself reflects a non-stationary characteristic of the narrowband data. As such, narrowband entropy appears to capture the difference between stationary power and the regularity of bursts, providing evidence for a potential dissociation between the two. Given that these indices relate in this application, it is hard to determine whether narrowband MSE *per se* adds information over and above burst analysis. The current evidence only allows us to discuss a potential dissociation from stationary power estimates. In theory however, narrowband entropy should also capture narrowband amplitude fluctuations, as for example investigated using detrended fluctuation analysis.

As stated in response to Reviewer 1, we have added a discussion on the benefits and disadvantages of a narrowband approach w.r.t broadband effects (see lines 929ff.). This hopefully clarifies the relation to the traditional ‘broadband’ sensitivity obtained with traditional estimates.

Some other questions came to mind about this narrowband MSE measure:
It seems very likely the estimate of MSE calculated from bandpassed data would be dependent on the width of the bandpass filters applied. Is this the case? And if so, what does this relation mean, in practice, for computing this measure, and how should one choose bandwidths?
Narrow bandpass filters (and the bandpass filters applied here seem quite narrow, at the lower frequencies) enforce sinuisoidality onto the filtered data. Narrowband filtering then removes what may be considered interesting waveform shape properties that one might have considered could be an interesting driver of entropy differences. This seems potentially related to the notion that this form of MSE may not capture some desired notions of signal irregularity. The question is then: how does the narrowband filtering effect waveform shape in a way that might affect entropy measures?
Should one consider potential differences in centre frequency if one is comparing between groups? The two groups in this analysis differ in age in a manner that would be expected to demonstrate a difference in peak alpha frequency. Given this, could or should one consider aligning compared bands to individual centre frequencies? If not, to what extent might difference in entropy simply relate to differences in centre frequency?
To be clear, I think that a full investigation of all the properties of the bandpass filter version of MSE could quickly become large, and that is out of scope of this particular manuscript. I don't consider that these 'other questions' must all be addressed or answered in this manuscript. They are suggested to motivate that it is perhaps worth adding a quick discussion point noting that there are open questions (and that there may be some limitations) to the narrowband MSE approach, and that further work is needed on this.

We discuss the loss of non-sinusoidal features (which spectrally would be represented as multi-scale coupling) in original submission of our manuscript: “However, this sensitivity trades off with specificity when a narrowband approach is chosen, which by definition enforces a more rhythmic appearance than the raw signal may convey (Cole & Voytek, 2018).” (lines 852ff.)

Regarding the reviewer’s point that “Given this, could or should one consider aligning compared bands to individual centre frequencies? If not, to what extent might difference in entropy simply relate to differences in centre frequency?” we agree that this is an interesting proposal. As eBOSC’s estimates are by default specific to individual frequencies, the overlap between eBOSC and MSE results is encouraging. But more work is needed to investigate the reviewer’s specific question and whether potential biases arise from mismatched frequencies. More generally, we believe that the question whether specific filter settings are optimal will depend on specific use cases. In the current manuscript, we feel it is important to highlight generally that spectral control is a potential avenue for ensuring time-scale specificity.

We added discussion of further work on establishing boundaries of narrowband MSE, it’s limitations and potentials. Furthermore, we stated that further analyses need to establish the adequacy of using identical frequency bands:

“However, further work is required to establish the functional interpretation of narrowband age differences, as well as technical impacts of filter bandwidth, and individual center frequencies on narrowband results, especially given age differences in rhythmic peak frequencies (Ishii et al., 2017).“ (lines 843ff.)

2) Simulating Variable 1/f

If there is one asymmetry between what is done in the simulations and what is presented in the real data, I would say it is that a key point made in the EEG data is how the difference in 1/f backgrounds of the data is a key factor in affecting MSE measures, and yet this point is not demonstrated in the simulated data. All of the simulations use a single value for x in 1/f^x. While I think the inference and interpretation of the 1/f effects are clear, if it is relatively straight-forward to compute and add, a set of simulations across different 1/f components of the simulated data (perhaps just 2 different values for x, representing each group) might be a nice addition. This would clearly show, in the simulated data, that this effect can be isolated to changes in the 1/f structure of the data.

We have now added a supplementary simulation (Supplementary File 2; S7 Figure) of the impact of different 1/f slopes on MSE, highlighting scale-dependent cross-over effects that conceptually replicate previous simulations (Courtiol et al., 2016), but partially reflect biases due to fixed similarity bound. Using rescaled bounds, scale-free slope differences are indicated by broad scale entropy offsets without crossovers.

“This would clearly show, in the simulated data, that this effect can be isolated to changes in the 1/f structure of the data.”

Simulating the actual age differences is difficult, as early time scales reflect both the 1/f slopes as well as any narrowband events. However, we show that statistically accounting for interindividual differences in 1/f can account for the age difference at fine scales. This relation of fine-scale age differences to differences in PSD slopes is moreover in line with results by McIntosh, Kovacevic, and Itier (2008) (see also their supplement) in the context of adolescence.

We now also include a Supplementary surrogate analysis, which highlights that linear power differences are sufficient to produce the observed MSE effects in both the 'Original' MSE implementation, as well as our low-pass implementation.

3) Values for the PSD Slopes

A minor comment, for clarification, is regarding the actual values for the PSD slopes that are plotted, for example in Figure 6 D1 and in Figure S6. The actual values are very small, and not typical of values for PSD slopes, which are typically closer to values of -1 (equivalent to 1/f^1, or pink noise). A 'back of the envelope' estimated calculation of the slope of the spectrum plotted in Figure 6 A2 is also more consistent with this value or around -1. Have the potted numbers been transformed in some way? I found no mention in the methods that would explain this, and so some clarification of the values plotted would be useful. Perhaps related, it is unclear why the words 'minus 0' appear in the title of the panel D1.

The reviewer correctly notes that the fit values in log-log space should be close to -1 in our dataset. However, for the previous fits, we performed a log(power)-frequency fit, not a log(power)-log(frequency) fit. Repeating the analysis with log-log fits indeed indicates values of around -1, with high inter-individual consistency with log-normal estimates (not shown). As the linearity increases for the log-log fit, and to increase consistency with the log-log slopes shown in the Summary Figure (now Fig 12), we repeated all relevant analyses with log-log fits and replaced the corresponding analyses. This applies to Figure 6 and 9, as well as the section "Low-frequency contributions render fine-scale entropy a proxy measure of PSD slope" and Figure S6. This did not qualitatively change the results.

Minus 0 (comparison to baseline) indicated a contrast of slopes against zero, which arguably is of limited information as no positive or zero slopes would be expected. We have therefore removed this comparison and only retain the visualization of average slope values in the figure.

4) Figure Tweaks

Finally, I have is actually a collection of notes and small suggestions for the figures. There are a lot of figures which nicely present a lot of data and findings, but I feel like some small updates would help make the figures a little more accessible to the new reader.

Figure 1
In 1B, the 'similarity bound' is indicated as '(r x SD)', where SD presumably standards for standard deviation. However, in the figure legend, similarity bounds are defined as 'r * 0.5 of signal variance'. It would be clearer if one form of the definition was used, which based on the other figures, I presume should be the one using standard deviation. It would be useful to define 'SD' here.
Figure 2
It's a small aesthetic nitpick: but the y-axis labels for A & B are not aligned
Figure 4
I think it's a mistake that the legend refers to a 'blue-to-red' line gradient?
Fig 6:
I initially found it slightly unclear on how to read and interpret the contrasts and topographies. If the paper is to be organized with the methods at the end (as current), I think it would be useful to note in the figure legend a brief note on what the p-values are that are plotted (these are presumably the results of the significance of the cluster-level statistic?)

The legend suggests asterisks are used to indicate significance, but this does not appear to be used in the plot?
I initially found the labelling to be unclear and wasn't sure what the 'B1', 'C1', etc labels were in panels 'A1' and 'A2'. Assuming these refer to the other sub-panels (and seem to motivate the labelling) briefly noting what these refer to in the legend could be useful.
Is A1 the 'Original' measure of entropy? This should indicated in the title and/or legend.
Figure 7:
I'm unclear on why the x-scale for Row 1 appears to be different from how it was plotted in Figure 6A1. Is what is plotted in Figure 6A1 not a copy of one of the plots in Figure 7 Row 1? It would be easier to highlight this if the scale were the same. Perhaps there is a reason they are different, but this could be noted, as otherwise it initially make the plotted measures between figure seem more different than they actually are.
Could you label the rows? In particular, I found it somewhat unclear as to what comparisons rows 2 and 4 were exactly, and especially row 4 (I believe it is the difference of similarity bounds, between groups, across channels?). This could be indicated more clearly in the figure legend.
It would be useful to you indicate (probably in the legend) in which direction the comparison was made, so that the direction of the t-values can be interpreted with respect to groups.
Figure 8:
The title in C says that entropy difference is <8 Hz, but the figure legend reports it's < 6 Hz.
Figure 9:
The labels for the lines in B are not clearly differentiated. The long dash and full solid labels look the same in the way they are plotted in the legend.
Figure 10:
Are all the topographies on the same color bar? The color bar is not consistently plotted, and if one is to be used across all topographies, this could be noted in the legend.
For G & H, are these collapsed across age groups? This would be useful to clarify.
Figure 11:
A stylistic suggestion is that I don't think you need the 'higher frequencies' arrow twice (at both the top and the bottom).
I initially found it somewhat unclear as to how to interpret the dashed coloured arrows. I'm not entirely sure if there is a change to be made, though perhaps they could be briefly mentioned in the figure legend, for clarification?

We thank the reviewer for these thorough comments and hope that we have successfully implemented some many? of the suggestions to improve the presentation of our Figures.

Figure 1: We now use standard deviation in the figure caption.

Figure 2: The y-axis labels are now aligned.

Figure 4: This was indeed a mistake that has now been fixed.

Figure 6: The figure contrast compared slopes against zero, which arguably is of limited information as no positive or zero slopes would be expected. We have therefore removed this comparison and only retain the visualization of average slope values. We now note in the figure caption that "*P-values correspond to the two/sided significance test of the cluster-level statistic*". Asterisk presentation has been replaced by a clearer visualization using yellow dots now. We now note the correspondence of the inset labels in A1/2 to the topographies in panels B/C. The heading of A1 now specifies that data result from the 'Original' entropy algorithm.

Figure 7: Figure 6A presents the same content as Figure 7A1, but with a log-scaled x-axis to emphasize the fine-scale differences. We have added a note to the legend of Figure 6A to clarify this. We extended our description of the rows to highlight the measure and direction of the contrast shown in rows 2 and 4. "(I believe it is the difference of similarity bounds, between groups, across channels?)" Row four indeed presents the group difference of similarity bounds, but by channel (whereas row 3 indicates the across-channel average).

Figure 8: We thank the reviewer for spotting this typo in the legend. Data In panels C and D were averages below 8, not 6 Hz. The figure titles were correct.

Figure 9: We thank the reviewer for spotting this formatting error. We have now fixed the visual legend differentiation.

Figure 10: We added the colorbar information to the Figure legend. We also added the information that the alpha and beta visualizations have been collapsed across groups.

Figure 11 (now Figure 12): We have retained the 'higher frequencies' arrow to highlight the increasing sensitivity to higher frequencies at finer scales. We have added a note to the figure caption highlighting that the dashed colored arrows indicate the mismatched relations observed in the current study.

Reviewer #3:

This is a comprehensive and thought-provoking assessment of the relation between spectral power (SP) and multiscale entropy (MSE), both of which have been used to characterize changes in brain dynamics across a wide range of conditions (mainly from EEG data). One of the perpetual challenges with MSE is the attempt to relate the scales for downsampling to something akin to frequency. This has some support in that there does appear to be some similarity in scale differences in MSE and frequency differences in SP. For example, the authors show age-related differences in MSE (higher fine-scale, lower coarse-scale) map to SP changes with higher power in faster frequencies and lower at slower frequencies - similar to what we (McIntosh) and others have published. While it is tempting to link these two metrics, I think most agree that it is not so straightforward (figure 11 sort of suggests this).

The first point that is emphasized is that the "original" MSE algorithm does not change the similarity criteria with successive downsampling of the signal. This appears to introduce a bias at coarse scales. This is a concern and definitely needs to be considered for MSE applications. The authors quite convincingly show that this can affect interpretations of group differences (Fig 7).

We thank the reviewer for highlighting this aspect of the manuscript. The general mechanism of this link suggests that this issue not only affects interpretations of group differences, but also of individual differences.

The second point, if I understand correctly, is that a direct mapping between scale and frequency is difficult because of cross-spectral dependencies. This isn't too surprising and in my read of the extant literature, I think most would agree. The Courtiol paper, for example, does make this point, and most of the work we did notes the similarities in MSE and SP, but also that they are sensitive to different aspects of brain signals. For example, nonlinearities (e.g., cross-spectral dependencies) do affect MSE, but by definition do not affect SP. Indeed,

the surrogate analysis the authors mention (which was done in the McIntosh et al, 2008 paper), shows this quite nicely. Also some other work (Bruce, Bruce, Vennelaganti, 2009) suggested that better predictor of sample entropy was found to be related to the power ratio of higher to lower frequencies:(alpha + beta)/(delta + theta). Moreover, the direct link between distance (local vs distributed), SP and MSE is also not straightforward. For example, while the age-effects reported in the present paper and by others do replicate nicely, there does appear to be a age-related change in local entropy (higher in ageing) and mutual information (lower in ageing), which maps to coherence estimates (McIntosh et al, Cereb Cortex 2014). These "connectivity" effects, however, are not constrained to a particular scale or frequency, which emphasizes the inherent nonlinearity in these effects. The Courtiol et al work also notes this sensitivity to nonlinearities.

"The second point, if I understand correctly, is that a direct mapping between scale and frequency is difficult because of cross-spectral dependencies." Our argument is more general than cross-spectral dependencies and concerns the fact that the interpretation of time scales needs to include an assessment of the spectral profiles *in general*. Given that faster processes are characterized by activity at higher frequencies, a natural interpretation may be that scale-specific content is more sensitive to the frequency content at this scale. However, due to the exclusive use of low-pass filters in the MSE computation, there has been no assessment of 'high-frequency'-specific entropy in most extant work. We see no principled argument against applying an alternative MSE algorithm, where scale-wise high-pass filters are applied – as done in our implementation – to probe irregularity of signals after excluding slow fluctuations.

"The Courtiol paper, for example, does make this point, and most of the work we did notes the similarities in MSE and SP, but also that they are sensitive to different aspects of brain signals. For example, nonlinearities (e.g., cross-spectral dependencies) do affect MSE, but by definition do not affect SP. Indeed, the surrogate analysis the authors mention (which was done in the McIntosh et al, 2008 paper), shows this quite nicely." While we agree with MSE's partial sensitivity to non-linear signal characteristics, we argue that this is often an unchecked assumption in empirical data. This is problematic, as MSE and spectral power are jointly sensitive to linear features (such as autocorrelations). For example, some work conceptually evokes non-linear contributions to MSE effects by highlighting that relative narrowband power (obtained by division with total power) does not show similar group differences as MSE (Takahashi et al., 2010). While convincing at face value, our results suggest that this is an inadequate procedure to identify non-linear effects, given MSE's sensitivity to linear broadband phenomena (PSD slopes). [This is true especially in combination with the similarity bias that we highlight as "Issue 1"] We agree that surrogate analysis is an optimal approach to identify unique non-linear contributions to MSE results. Interestingly, in McIntosh et al, 2008, surrogate analysis did not indicate any fine-scale differences (which could be recovered by simulating spectral slope variations) and only indicated coarse-scale differences between the original and the shuffled signals. However, this supplementary analysis did not compare age differences which were the main contrast in the paper. As such, it is unclear to what extent age differences at coarser scales reflected valid age variations in either (a) non-linearity and/or (b) specifically slower signals, especially given the issue with fixed similarity bounds noted in our work and by others previously (which may also affect the results presented in McIntosh et al. (2008, PLOS CB)).

"Also some other work (Bruce, Bruce, Vennelaganti, 2009) suggested that better predictor of sample entropy was found to be related to the power ratio of higher to lower frequencies:(alpha + beta)/(delta + theta)." This sensitivity is reasonable given that fine-scale sample entropy is highly sensitive to aperiodic slopes, which may strongly contribute to such power ratios. Recent

work from Donoghue, Dominguez, and Voytek (2020) shows this in a principled way. This is further in line with the observation that entropy (multiscale dispersion entropy in this case) covaries with spectral slopes across sleep stages (Miskovic, MacDonald, Rhodes, & Cote, 2019). We highlight this aspect throughout the manuscript (see section *Low-frequency contributions render fine-scale entropy a proxy measure of PSD slope*; see novel section *Alpha events transiently reduce broadband signal irregularity* for an empirical argument to dissociate narrow- and broadband content) and have cited this work in our previous submission: "This conceptual link between PSD slopes and sample entropy has been empirically observed both across subjects and wakefulness states (Bruce, Bruce, & Vennelaganti, 2009; Miskovic et al., 2019; Waschke, Wostmann, & Obleser, 2017)." The reviewer is correct that this description is imprecise as Bruce et al. do not directly show an association with slopes and we inferred potential relations based on the above rationale. We have therefore adjusted this statement as follows: "This conceptual link between PSD slopes (or high-to-low frequency power ratios that may have strong broadband slope contributions (Donoghue et al., 2020)) and sample entropy has been empirically observed both across subjects and wakefulness states (Bruce et al., 2009; Miskovic et al., 2019; Waschke et al., 2017)." (lines 185 ff.).

"There does appear to be a age-related change in local entropy (higher in ageing) and mutual information (lower in ageing), which maps to coherence estimates (McIntosh et al, Cereb Cortex 2014)." Here, we exclusively assessed univariate entropy, and did not consider the conditional entropy or mutual information between bivariate time series as done in McIntosh et al, Cereb Cortex 2014. While we have not tested this, a reduction in entropy between sensors may also relate to lower-order PSD features. McIntosh et al. (2014) features a supplementary spectral power analysis for the univariate MSE analysis, but not for the mutual information analysis. As such, their potential dissociability remains unclear, but poses an interesting question. This directly relates to the question of how higher-order dependencies such as covariations, depend on lower order estimates, such as simple power or univariate entropy. We now briefly note this as a future direction: "It is an interesting question for future work, whether similar biases contribute to age-related decreases in 'distributed' entropy that captures the mutual information between distinct sensors (McIntosh et al., 2014)."

"These "connectivity" effects, however, are not constrained to a particular scale or frequency, which emphasizes the inherent nonlinearity in these effects." It is unclear to us why the reviewer assumes that effects across multiple scales, especially in implementations with fixed bounds, unambiguously indicate nonlinear effects. As we highlight, linear PSD slopes produce effects across multiple entropy 'time scales', as would the mere variation of (uncoupled) oscillations. In general, the scale-wise low-pass filtering only decreases the sensitivity to fast events at fine scales (even this does not hold when fixed similarity bounds are used). Thus, at present, we feel that the unambiguous inference of nonlinear contributions from observing multi-scale effects is not warranted. However, the reviewer makes a more general argument regarding entropy's potential sensitivity to non-linearities that we agree with (see next point).

Thus, I am left wondering if the second message is as conclusive as the authors seem to suggest. It's complicated, no doubt, as the Venn diagram in Figure 11 suggests. Which takes me to the main point. I get the impression that the authors advocate SP as the 'gold standard' on which to map the MSE effects, but I am not sure this is a valid. The simulation used to support this changes power at the alpha frequency in an 1/f signal, which makes a very strong assumption on the underlying biological processes. Thus, insofar as the brain signals actually do this -- add power to a restricted frequency band -- the simulation is useful. But, if there are changes in the brain signal that span frequencies (e.g., cross-spectral dependencies), then the spectral filters won't really help and may in fact obscure things. I suggest the authors actually

do the surrogate analyses they propose as suggestion (d) on page 32 to really disentangle this. Otherwise, I am concerned that someone who reads this will come away thinking that measuring SP is really all we need to do, which I don't think is the message the authors want to convey.

"I get the impression that the authors advocate SP as the 'gold standard' on which to map the MSE effects, but I am not sure this is a valid." We have two major responses to this impression. First, we do not intend to argue that spectral power is a gold standard to describe neural dynamics. Even in the case of narrowband events, spectral power can be an imperfect index due to its assumption of sinusoidal stationarity – as argued by our use of narrowband rhythm detection. Rather, we focus on the mapping between MSE and spectral power, as the period or frequency of signal fluctuations are perhaps among the only rigorous descriptors of time scales (MSE does not permit informative characterizations about specific time scales at present). We have now changed the title accordingly to specify that the characteristic of interest is the time scale of neural dynamics, not its estimation using power (see also comment to Reviewer 1). If specificity of time scale is not of interest, then the utility of MSE over simple SE is immediately called in question, as the latter already captures broad-scale effects. The simulation of frequency-specific, periodic events, allows us to assess such intuitive interpretations regarding MSE time scales. Second, we suggest that entropy's sensitivity to a multitude of signal characteristics increases the need to identify contributors to specific effects to make informative claims about brain function (at interpretable time scales). The theoretical sensitivity to broadband effects and non-linearities is a major advantage of sample entropy (and thus MSE) over traditional, linear, spectral approaches; however, we argue that circumventing MSE's biases and better post-hoc spectral control of identified effects may go a long way to ensure that identified effects are truly due what might be attributable to signal non-linearities.

"I suggest the authors actually do the surrogate analyses they propose as suggestion (d) on page 32 to really disentangle this." We agree with the reviewer that our mention of surrogate analyses without actually implementing them was a drawback of our previous submission. We have now rectified this and added a surrogate analysis (see Supplementary file 3, S9 Figure). This analysis highlights that linear contributions are sufficient to capture the observed main age effects. This matches our statistical control analyses of the two main age effects: (a) a fine-scale entropy increase for older adults, that can be explained by differences in aperiodic slopes (as also observed in McIntosh et al, 2008 and others, and cited in the main manuscript); and (b) a decrease in coarse-scale entropy that can be accounted for by high-frequency power differences due to the use of fixed similarity bounds. Our results further agree with the conclusions by Courtiol et al. (2016) who employed surrogate analysis and concluded that MSE age differences at rest mainly relate to linear PSD properties (see e.g., Figure 7C in their paper). However, we agree with the reviewer that the contributions to MSE exceed the linear ones, which we also show with a deliberate test for linearity using surrogate ratio scores. Notably, visual inspection of MSE ratios (S9 Figure D,E) hints at potential age differences at fine- and coarse-scales that do not exceed statistical significance criteria however, and at best make small contributions to the MSE age differences obtained in the original data without surrogate control. The similarity between surrogate ratio scores for different implementations underline the notion that surrogate analyses provide a powerful tool to identify non-linearities even in the presence of linear power differences and methodological power biases. We remain convinced that the use of surrogates will prove important for investigating non-linear brain dynamics in future work.

"Otherwise, I am concerned that someone who reads this will come away thinking that measuring SP is really all we need to do, which I don't think is the message the authors want to convey." We indeed hope that this is not the take-away message from our work. Instead, we

hope to offer a constructive critique and highlight specific controls that "may go a long way toward establishing unique, complementary, and valid contributions of MSE in future work" (see *Recommendations for future applications*). Moreover, our conclusions summarize our perspective that "[…] MSE effects may be taken as a starting point to explore the linear and nonlinear features of brain signals (e.g., Simpraga et al., 2017). We believe that empirical identification of the unique predictive utility of MSE will advance the quest for reliable mechanistic indicators of flexible brain function across the lifespan, and in relation to cognition, health, and disease." In some scenarios, linear properties may suffice to capture entropy effects. In these scenarios, measuring SP is indeed all we need to do, and we argue that a parallel literature with less precision and more ambiguous interpretations should be avoided. More interesting scenarios emerge for MSE as a metric if this can be avoided. Our work suggests best practices to identify such interesting scenarios, in which non-linearities may strongly contribute to obtained MSE estimates, with the hope that this advances our understanding and measurement of neural computations.

Reviewer #4:

This is a well-done paper. The authors should be congratulated for their thorough and didactic exposition of an intricate problem. However, the study left me unconvinced that, at least in its current state, it has a place in a leading computational neuroscience/biology journal. I will list my concerns in more detail below, but will have to leave it to the editors to weigh my comments against the broader context of the scope of their journal.

The paper starts out with multi-scale entropy (MSE) being a key measure, "increasingly", "often", "commonly" applied in a specific way, which the authors go on to explain in admirable detail (i.e., applying a broad-band variance scaling). The authors then go on to show how this creates a severe band-specific spectral power confound. Using cross-sectional data from young and older brains they go on to argue that conclusions on entropy/variability changes over the life-span could as well be confounded by power. They end on a set of cook-book-style recommendations. I have not much to quibble with in the way they set out this argument.

We thank the reviewer for indicating that our line of argumentation is clear and convincing.

The overriding impression, however, was that of a data analysis tutorial or a methods paper. The implications for our understanding of neural functioning and/or neuro-cognitive computation did not become clear.
From the outset, I had been expecting a closer link to the neurobiology of (multi-scale) entropy, and I do think that such a link is necessary to lift the current paper, and its potential impact, beyond the ranks of a methodological comment.

Not least, the mixture of simulations and data re-analysis left me furthermore unclear what I am to take from this study. For a hardcore signal analysis/digital signal processing audience, some of the treatment might be too shallow, while for the applied neuroscientist the particular example of age-related changes in spectral slope might be too specific to fully take away what the consequences for future MSE applications might be.

Should a revision be invited, I would suggest to focus the paper more on what we learn about age-related change in the spectral composition / E:I imbalance in the aging brain and would

suggest to tone down the overly specific, cook-book-like recommendations on how to analyse MSE instead.

”The implications for our understanding of neural functioning and/or neuro-cognitive computation did not become clear.” We believe that our understanding of neural functioning will be advanced only once we can measure signatures of this computation, and be able to validly interpret what these signatures may reflect at the computational level. A major part of this is correctly identifying the time scale (or the broadband nature) of observed effects as the very nature of neural time scales/frequencies is at the heart of much of the current neurophysiological literature, theory, and computational modeling. More broadly, a major goal here is to identify the features that contribute to differences in signal irregularity as measured in non-invasive scalp recordings. In applications, the use of MSE is often motivated by highlighting its unique sensitivity (over traditional PSD applications) to non-linear signal characteristics, invoking arguments for dynamic systems views of neural function, etc. (see also comments above). However, these portrayals neglect that this relative sensitivity may be low relative to linear signal characteristics. As such, we believe that our results are of crucial importance for identifying non-linear contributions in future work. More generally, we believe that partial dissociations of these contributors to global brain dynamics will advance substantive questions regarding neural computations.

We agree with the reviewer that by highlighting general methodological issues, our previous submission was light on neurobiological interpretation. We have attempted to rectify this in the revision and have added more extensive discussions relating to potential neurobiological interpretations (*Issue 2: Fine-scale entropy relates to PSD slopes in the presence of slow frequency content*) as well as potential sources of age differences (*Relevance of identified time scale mismatches to previous work*). Moreover, we have added a novel analysis that highlights the utility of sample entropy for assessments of spontaneous neural dynamics (*Rhythmic alpha events transiently reduce broadband signal irregularity*), especially when the principles highlighted in our manuscript are considered. We argue that this example highlights how sample entropy – if its limitations are considered – may advance our understanding of neural computations, by indicating a non-trivial, association between non-stationary alpha power events and broadband sample entropy. While our paper does not aim to show an exclusive list of entropy’s sensitivities, we believe that our methodological advances will inform future applications to – in turn – advance neuro-computational insights.

“For a hardcore signal analysis/digital signal processing audience, some of the treatment might be too shallow, while for the applied neuroscientist the particular example of age-related changes in spectral slope might be too specific to fully take away what the consequences for future MSE applications might be.” We thank the reviewer for sharing this impression. We indeed hope that the treatment is widely accessible to applied neuroscientists and (entropy) signal analysts alike, which judging by the encouraging reviewer comments, seems to have been achieved. We would like to highlight two points with regard to the reviewer’s second statement that our results may be too specific for implications to be extracted by applied neuroscientists. First, the reviewer acknowledges and focuses on the second issue of broadband contributions to local scales, but did not refer to the first, in our view more severe issue of fixed similarity bounds. This issue may *completely invalidate* the prevalent (and intuitive) interpretation of *any* coarse-scale result obtained with fixed similarity bounds. To clarify the practical relevance of this bias, we use a conceptual replication of past work on adult age differences. We have now further emphasized this point in the discussion to clarify the practical consequences. The second issue is indeed a conceptual one that aims at the general broad-scale nature of local scale information, as well as the need to define signatures of interest, which is generally not done in the extant literature. In particular, the link to 1/f slopes highlights that in

this scenario, non-linearities contribute little to the observed age effects, although the theoretical sensitivity of entropy to non-linearities remains a major use case for its adoption. From a different perspective, this sensitivity to broadband spectral characteristics can also be of importance for neuroscientific questions, but only if circularity issues are avoided. We hope that our added use case of alpha-broadband entropy coupling is revealing in this regard. As such, we wish to highlight *general* principles that need to be considered to advance potential insights into neuro-cognitive computations.

"I would suggest to focus the paper more on what we learn about age-related change in the spectral composition / E:I imbalance in the aging brain and would suggest to tone down the overly specific, cook-book-like recommendations on how to analyse MSE instead." We respectfully disagree with the reviewer regarding the cook-book style of the discussion. We decided to retain the 'cook-book' approach to our final recommendations as we believe that these practical takeaways may ultimately prove the most useful for readers to understand how and why MSE may not work as they believe it does, especially when applying MSE in a standard manner (e.g., with fixed similarity bounds). The current state of the literature would suggest that the majority of regular users of MSE do not appreciate how incorrect their inferences may be (e.g., with regard to time scales). Such incorrect inferences impact the veracity of all conclusions about neural computation such studies may offer. As such, we feel that communicating key issues clearly and with reasonable depth is what makes our paper uniquely useful.

However, we fully agree with the reviewer that we could better highlight and discuss our observed age effects. On page 22f., we extended our discussion of broadband spectral slopes, citing evidence suggesting that broadband power may reflect the overall level of neural output. Moreover, on page 24 we have extended our discussion of two differential perspectives of age-related changes in spectral slopes and entropy:

"Across development, altered time scales of neural computations (as indicated by broadband changes in autocorrelations) (Murray et al., 2014) may reflect changes in intra- and inter-cortical connectivity (Duarte, Seeholzer, Zilles, & Morrison, 2017), arising from reductions in grey matter density (Raz et al., 2005; Sowell et al., 2003), the integrity of associative white matter tracts (Bender, Volkle, & Raz, 2016), and changes in local receptor distributions and neuromodulation (Hua, Kao, Sun, Li, & Zhou, 2008; Lalwani et al., 2019; Leventhal, Wang, Pu, Zhou, & Ma, 2003; Schliebs & Arendt, 2011; Tatti, Haley, Swanson, Tselha, & Maffei, 2017). Dynamic interactions between such morphological changes may jointly shape control over local excitability and 'neural noise' across the lifespan (Li & Rieckmann, 2014). Two alternative functional consequences of developmental noise increases have been proposed. On the one hand, intermediate levels of noise may provide beneficial stochastic resonance effects (Garrett, McIntosh, & Grady, 2011; McDonnell & Ward, 2011; Mcnamara & Wiesenfeld, 1989; Wiesenfeld & Moss, 1995), in line with relations between higher entropy and behavioral benefits in child- and adulthood (McIntosh et al., 2008), as well as in older adults (Heisz, Gould, & McIntosh, 2015). In contrast, overwhelming amounts of local noise can produce adverse consequences (MacDonald, Nyberg, & Backman, 2006; Voytek et al., 2015), supported by the observation that shallower slopes with advanced adult age relate to impaired working memory performance (Voytek et al., 2015). While further work including longitudinal assessments and behavioral probes will be necessary to disentangle the functional relevance of developmental changes, we argue that a principled separation of

narrow- and broadband changes (Voytek & Knight, 2015) will help to guide the search for neurobiological mechanisms driving entropy effects." (lines 809ff.)

Minor: SD and variance are used a bit too interchangeably, especially around Fig. 1/caption thereof.

We thank the reviewer for indicating this discrepancy, which mirrors a comment from Reviewer 2. We have now reduced the use of variance, and focused on using standard deviation.

References

Bender, A. R., Volkle, M. C., & Raz, N. (2016). Differential aging of cerebral white matter in middle-aged and older adults: A seven-year follow-up. *Neuroimage, 125*, 74-83. doi:10.1016/j.neuroimage.2015.10.030

Bruce, E. N., Bruce, M. C., & Vennelaganti, S. (2009). Sample Entropy Tracks Changes in Electroencephalogram Power Spectrum With Sleep State and Aging. *Journal of Clinical Neurophysiology, 26*(4), 257-266. doi:10.1097/WNP.0b013e3181b2f1e3

Cole, S., & Voytek, B. (2018). Cycle-by-cycle analysis of neural oscillations. *bioRxiv*.

Courtiol, J., Perdikis, D., Petkoski, S., Muller, V., Huys, R., Sleimen-Malkoun, R., & Jirsa, V. K. (2016). The multiscale entropy: Guidelines for use and interpretation in brain signal analysis. *Journal of Neuroscience Methods, 273*, 175-190. doi:10.1016/j.jneumeth.2016.09.004

Donoghue, T., Dominguez, J., & Voytek, B. (2020). Electrophysiological Frequency Band Ratio Measures Conflate Periodic and Aperiodic Neural Activity. *bioRxiv*.

Duarte, R., Seeholzer, A., Zilles, K., & Morrison, A. (2017). Synaptic patterning and the timescales of cortical dynamics. *Current Opinion in Neurobiology, 43*, 156-165. doi:10.1016/j.conb.2017.02.007

Faes, L., Pereira, M. A., Silva, M. E., Pernice, R., Busacca, A., Javorka, M., & Rocha, A. P. (2019). Multiscale information storage of linear long-range correlated stochastic processes. *Physical Review E, 99*(3). doi:ARTN 032115
10.1103/PhysRevE.99.032115

Faes, L., Porta, A., Javorka, M., & Nollo, G. (2017). Efficient Computation of Multiscale Entropy over Short Biomedical Time Series Based on Linear State-Space Models. *Complexity*. doi:Artn 1768264
10.1155/2017/1768264

Garrett, D. D., McIntosh, A. R., & Grady, C. L. (2011). Moment-to-moment signal variability in the human brain can inform models of stochastic facilitation now. *Nature Reviews Neuroscience, 12*(10), 612; author reply 612. doi:10.1038/nrn3061-c1

Hardstone, R., Poil, S. S., Schiavone, G., Jansen, R., Nikulin, V. V., Mansvelder, H. D., & Linkenkaer-Hansen, K. (2012). Detrended fluctuation analysis: a scale-free view on neuronal oscillations. *Frontiers in Physiology, 3*. doi:ARTN 450
10.3389/fphys.2012.00450

Heisz, J. J., Gould, M., & McIntosh, A. R. (2015). Age-related Shift in Neural Complexity Related to Task Performance and Physical Activity. *Journal of Cognitive Neuroscience, 27*(3), 605-613. doi:10.1162/jocn_a_00725

Hua, T. M., Kao, C. C., Sun, Q. Y., Li, X. R., & Zhou, Y. F. (2008). Decreased proportion of GABA neurons accompanies age-related degradation of neuronal function in cat striate cortex. *Brain Research Bulletin, 75*(1), 119-125. doi:10.1016/j.brainresbull.2007.08.001

Ishii, R., Canuet, L., Aoki, Y., Hata, M., Iwase, M., Ikeda, S., . . . Ikeda, M. (2017). Healthy and Pathological Brain Aging: From the Perspective of Oscillations, Functional Connectivity, and Signal Complexity. *Neuropsychobiology, 75*(4), 151-161. doi:10.1159/000486870

Lalwani, P., Gagnon, H., Cassady, K., Simmonite, M., Peltier, S., Seidler, R. D., . . . Polk, T. A. (2019). Neural distinctiveness declines with age in auditory cortex and is associated with auditory GABA levels. *Neuroimage, 201*. doi:UNSP 116033
10.1016/j.neuroimage.2019.116033

Leventhal, A. G., Wang, Y. C., Pu, M. L., Zhou, Y. F., & Ma, Y. Y. (2003). GABA and its agonists improved visual cortical function in senescent monkeys. *Science, 300*(5620), 812-815. doi:DOI 10.1126/science.1082874

Li, S. C., & Rieckmann, A. (2014). Neuromodulation and aging: implications of aging neuronal gain control on cognition. *Current Opinion in Neurobiology, 29*, 148-158. doi:10.1016/j.conb.2014.07.009

Linkenkaer-Hansen, K., Nikouline, V. V., Palva, J. M., & Ilmoniemi, R. J. (2001). Long-range temporal correlations and scaling behavior in human brain oscillations. *Journal of Neuroscience, 21*(4), 1370-1377.

MacDonald, S. W. S., Nyberg, L., & Backman, L. (2006). Intra-individual variability in behavior: links to brain structure, neurotransmission and neuronal activity. *Trends in Neurosciences, 29*(8), 474-480. doi:10.1016/j.tins.2006.06.011

Mahjoory, K., Cesnaite, E., Hohlefeld, F. U., Villringer, A., & Nikulin, V. V. (2019). Power and temporal dynamics of alpha oscillations at rest differentiate cognitive performance involving sustained and phasic cognitive control. *Neuroimage, 188*, 135-144. doi:10.1016/j.neuroimage.2018.12.001

McDonnell, M. D., & Ward, L. M. (2011). The benefits of noise in neural systems: bridging theory and experiment. *Nature Reviews Neuroscience, 12*(7), 415-426. doi:10.1038/nrn3061

McIntosh, A. R., Kovacevic, N., & Itier, R. J. (2008). Increased Brain Signal Variability Accompanies Lower Behavioral Variability in Development. *Plos Computational Biology, 4*(7). doi:10.1371/journal.pcbi.1000106

McIntosh, A. R., Vakorin, V., Kovacevic, N., Wang, H., Diaconescu, A., & Protzner, A. B. (2014). Spatiotemporal Dependency of Age-Related Changes in Brain Signal Variability. *Cerebral Cortex, 24*(7), 1806-1817. doi:10.1093/cercor/bht030

Mcnamara, B., & Wiesenfeld, K. (1989). Theory of Stochastic Resonance. *Physical Review A, 39*(9), 4854-4869. doi:DOI 10.1103/PhysRevA.39.4854

Miskovic, V., MacDonald, K. J., Rhodes, L. J., & Cote, K. A. (2019). Changes in EEG multiscale entropy and power-law frequency scaling during the human sleep cycle. *Human Brain Mapping, 40*(2), 538-551. doi:10.1002/hbm.24393

Murray, J. D., Bernacchia, A., Freedman, D. J., Romo, R., Wallis, J. D., Cai, X. Y., . . . Wang, X. J. (2014). A hierarchy of intrinsic timescales across primate cortex. *Nature Neuroscience, 17*(12), 1661-1663. doi:10.1038/nn.3862

Nikulin, V. V., & Brismar, T. (2004). Comment on "Multiscale entropy analysis of complex physiologic time series". *Physical Review Letters, 92*(8). doi:10.1103/PhysRevLett.92.089803

Raz, N., Lindenberger, U., Rodrigue, K. M., Kennedy, K. M., Head, D., Williamson, A., . . . Acker, J. D. (2005). Regional brain changes in aging healthy adults: General trends, individual differences and modifiers. *Cerebral Cortex, 15*(11), 1676-1689. doi:10.1093/cercor/bhi044

Schliebs, R., & Arendt, T. (2011). The cholinergic system in aging and neuronal degeneration. *Behavioural Brain Research, 221*(2), 555-563. doi:10.1016/j.bbr.2010.11.058

Simpraga, S., Alvarez-Jimenez, R., Mansvelder, H. D., van Gerven, J. M. A., Groeneveld, G. J., Poil, S. S., & Linkenkaer-Hansen, K. (2017). EEG machine learning for accurate detection of cholinergic intervention and Alzheimer's disease. *Scientific Reports, 7*. doi:10.1038/s41598-017-06165-4

Sowell, E. R., Peterson, B. S., Thompson, P. M., Welcome, S. E., Henkenius, A. L., & Toga, A. W. (2003). Mapping cortical change across the human life span. *Nature Neuroscience, 6*(3), 309-315. doi:10.1038/nn1008

Takahashi, T., Cho, R., Mizuno, T., Kikuchi, M., Murata, T., Takahashi, K., & Wada, Y. (2010). Antipsychotics reverse abnormal EEG complexity in drug-naive schizophrenia: A multiscale entropy analysis. *International Journal of Neuropsychopharmacology, 13*, 242-243.

Tatti, R., Haley, M. S., Swanson, O. K., Tselha, T., & Maffei, A. (2017). Neurophysiology and Regulation of the Balance Between Excitation and Inhibition in Neocortical Circuits. *Biological Psychiatry, 81*(10), 821-831. doi:10.1016/j.biopsych.2016.09.017

Valencia, J. F., Porta, A., Vallverdu, M., Claria, F., Baranowski, R., Orlowska-Baranowska, E., & Caminal, P. (2009). Refined Multiscale Entropy: Application to 24-h Holter Recordings of Heart Period Variability in Healthy and Aortic Stenosis Subjects. *Ieee Transactions on Biomedical Engineering, 56*(9), 2202-2213. doi:10.1109/Tbme.2009.2021986

Voytek, B., & Knight, R. T. (2015). Dynamic Network Communication as a Unifying Neural Basis for Cognition, Development, Aging, and Disease. *Biological Psychiatry, 77*(12), 1089-1097. doi:10.1016/j.biopsych.2015.04.016

Voytek, B., Kramer, M. A., Case, J., Lepage, K. Q., Tempesta, Z. R., Knight, R. T., & Gazzaley, A. (2015). Age-Related Changes in 1/f Neural Electrophysiological Noise. *Journal of Neuroscience, 35*(38), 13257-13265. doi:10.1523/Jneurosci.2332-14.2015

Waschke, L., Wostmann, M., & Obleser, J. (2017). States and traits of neural irregularity in the age-varying human brain. *Scientific Reports, 7*. doi:10.1038/s41598-017-17766-4

Wiesenfeld, K., & Moss, F. (1995). Stochastic Resonance and the Benefits of Noise - from Ice Ages to Crayfish and Squids. *Nature, 373*(6509), 33-36. doi:DOI 10.1038/373033a0

Xiong, W. T., Faes, L., & Ivanov, P. C. (2017). Entropy measures, entropy estimators, and their performance in quantifying complex dynamics: Effects of artifacts, nonstationarity, and long-range correlations. *Physical Review E, 95*(6). doi:ARTN 062114
10.1103/PhysRevE.95.062114
